# Heterogeneity of Extracellular Vesicles and Non‐Vesicular Nanoparticles in Glioblastoma

**DOI:** 10.1002/jev2.70168

**Published:** 2025-10-02

**Authors:** Tuoye Xu, Joao A. Paulo, Piyan Zhang, Xinyue Liu, Alya Nguyen, Yuanhua Cheng, Clark Massick, Yanhong Zhang, Dennis K. Jeppesen, Qin Zhang, James N. Higginbotham, Oleg S. Tutanov, Anna M. Krichevsky, Daniel T. Chiu, Steve P. Gygi, Kasey C. Vickers, Jeffrey L. Franklin, Robert J. Coffey, Al Charest

**Affiliations:** ^1^ Cancer Research Institute Beth Israel Deaconess Medical Center Boston Massachusetts USA; ^2^ Department of Cell Biology Harvard Medical School Boston Massachusetts USA; ^3^ Department of Chemistry and Bioengineering University of Washington Seattle Washington USA; ^4^ Department of Medicine Vanderbilt University Medical Center Nashville Tennessee USA; ^5^ Department of Neurology Brigham and Women's Hospital and Harvard Medical School Boston Massachusetts USA; ^6^ Department of Cell and Developmental Biology Vanderbilt University School of Medicine Nashville Tennessee USA; ^7^ Department of Medicine Harvard Medical School Boston Massachusetts USA

**Keywords:** EGFR, exomeres, extracellular nanoparticles, extracellular vesicles, glioblastoma, supermeres

## Abstract

It is increasingly clear that intercellular communication is largely mediated by lipid‐bilayer, membrane‐bound extracellular vesicles (EVs) and amembranous, non‐vesicular extracellular particles (NVEPs), including exomeres and the recently identified supermeres. To elucidate the cargo and functional roles of these carriers, we performed a comprehensive analysis of their lipid, protein and RNA content in the context of colorectal cancer and glioblastoma (GBM). Our results demonstrate that EVs exhibit distinct density profiles correlated with specific biomolecular signatures. Moreover, EVs and NVEPs display notable differences in their protein and RNA composition, which confer distinct functional attributes. Supermeres are notably enriched in components involved in extracellular matrix remodeling and possess the ability to cross the blood–brain barrier, a process dependent on their intact structure and RNA content. Once in the central nervous system (CNS), they preferentially engage with microglia and suppress TGFβ1 expression, suggesting a role in modulating microglial immune activity. Furthermore, systemically administered exogenous supermeres selectively accumulate in GBM tumors in vivo. Together, these findings highlight supermeres as a promising vehicle for delivering therapeutics to the CNS and brain tumors.

## 1 Introduction

Glioblastoma (GBM) is a highly aggressive and incurable primary brain cancer that presents significant challenges in treatment, with median survival rates remaining dismal despite an intensive standard of care, which comprises surgical resection, radiation and chemotherapy. These measures only extend survival modestly (Fukushima and de Groot 2024). Amplification and mutations of the epidermal growth factor receptor (EGFR) are associated with approximately 70% of GBM cases (Brennan et al. 2013; McLendon et al. 2008; Verhaak et al. 2010). An intragenic rearrangement of EGFR, called EGFRvIII, is observed in 50% of EGFR‐positive cases and renders the receptor ligand‐independent and constitutively activated (An et al. 2018). Despite EGFR's proven driver mutation function in GBM (Zhu et al. 2009; Jun et al. 2012; Acquaviva et al. 2011), EGFR inhibition has remained ineffective in clinical settings (Lin et al. 2022).

One of the primary obstacles in treating GBM is its vast genomic and cellular heterogeneity (Boussiotis and Charest 2018). This diversity allows GBM cells to evade treatment by adapting and surviving in a variety of cellular states. Although a great deal has been uncovered regarding the genetic mutations associated with GBM (Dakal et al. 2024), much less is understood about how GBM cells interact with their surrounding tumor microenvironment, a critical aspect of cancer progression and treatment resistance. Effective communication between GBM cells and surrounding immune and support cells within the microenvironment enables the tumor to grow, evade immune detection and resist treatment. Understanding these interactions is increasingly recognized as essential for developing therapies that can disrupt these processes, highlighting the urgent need for further research in this area to identify novel strategies that could improve treatment outcomes for GBM patients.

EVs are phosphobilayer membrane‐bound particles released by cells, including cancer cells, into the extracellular environment (Mateescu et al. 2022). These vesicles contain bioactive molecules, such as DNA, RNA and proteins, which they transport between cells, playing a critical role in intercellular communication (Jeppesen et al. 2024). This transfer of molecules can affect various physiological and pathological processes, including disease progression (Mathieu et al. 2019). In recent years, EVs have attracted significant attention in cancer research due to their capacity to carry tumor‐derived biomarkers, which circulate in body fluids like blood, saliva and urine (Jeppesen et al. 2024). This accessibility makes EVs promising candidates for non‐invasive diagnostic tools and potential therapeutic applications (Welsh et al. 2024). For instance, recent developments in EV detection from biofluids demonstrate the feasibility of detecting phospho‐EGFR positive EVs from plasma samples of GBM patients (Maniya et al. 2024).

The heterogeneity within EV populations is illustrated by the wide range of functions they perform across different biological systems. This diversity in function and composition suggests that EVs are far from uniform, underscoring the need for more detailed characterization to fully understand their distinct roles and potential applications in health and disease. As the study of EVs has expanded, so has the field of intercellular communication, now including recently identified classes of amembranous non‐vesicular extracellular nanoparticles (NVEPs), such as exomeres and supermeres (Zhang et al. 2018, 2021).

Exomeres are a type of extracellular particle distinct from conventional EVs. Smaller than 50 nanometers, exomeres lack a phospholipid bilayer and are not formed through standard EV biogenesis processes, like budding or fusion of multivesicular bodies with the plasma membrane (Zhang et al. 2018, 2021). Rich in proteins, nucleic acids and other bioactive molecules like some lipids, exomeres have been linked to cellular signaling and processes related to cancer progression, such as cell adhesion, migration and immune response modulation (Zhang et al. 2018, 2021). Supermeres, an even smaller class of extracellular nanoparticles, share some physico‐chemical characteristics with exomeres, particularly in their lack of a phospholipid membrane and unique bioactive composition. Supermeres contain cancer signature proteins and are emerging as promising targets for biomarker discovery, diagnostic development and therapeutic strategies (Zhang et al. 2021). Here, we describe the functional and compositional differences across EVs and NVEPs, including EVs, exomeres and supermeres, isolated from GBM patient‐derived xenograft (PDX) cells and CRC cells based on multi‐omics approaches. Notably, we identified supermeres capable of penetrating the blood–brain barrier (BBB) and interacting with microglia both in vivo and in vitro, marking them as potential vehicles for central nervous system (CNS) therapeutic delivery. This study highlights the extensive heterogeneity of membrane‐bound EVs and NVEPs, paving the way for further exploration of their roles in disease and therapy.

## 2 Materials and Methods

### 2.1 In Vivo Biodistribution of EVs and NVEPs

15k EVs, 167k EVs, exomeres and supermeres isolated from human PDX GBM cell cultures GBM6, GBM39 and GBM59 and HEK‐293 and GL261 cells were labelled with near infra‐red (NIR) IRDye 800 CW NHS ester (LI‐COR, 929–70020) according to the manufacturer's protocol. The labelled 15k EVs were pelleted by centrifugation for 60 min at 10,000 g in a SW28 swing‐bucket rotor, and labelled 167k EVs were pelleted by centrifugation for 4 h at 167,000 g in a SW41 Ti swing‐bucket rotors. The labelled exomeres were pelleted by centrifugation at 167,000 g in a SW41 Ti swinging‐bucket rotor for 16 h. The supermeres were pelleted by centrifugation at 367,000 g using a Beckman Coulter SW55 Ti rotor for 16 h. The samples were resuspended and washed in PBS (pH 7.4) and then pelleted again. We experimentally determined that potential IRDye 800 CW unbound dye aggregates did not remain after the centrifugation and washing protocols used to purify labelled supermeres. Labelled samples (150 µg in 200 µL PBS) were injected intravenously by tail vein into 8‐week‐old male CD1 or C57Bl6/J mice. Their organs were harvested either 6 or 24 h after injection and imaged using the Odyssey imaging system (LI‐COR Biosciences). The average fluorescence intensity analysis of IRDye 800 accumulated in each organ was performed with the software Image Studio. All animal studies and procedures were approved by the Beth Israel Deaconess Medical Center Institutional IACUC (protocol no. 032–2025). Supermeres (150 µg of protein) were denatured at 100°C for 10 min or treated with RNase (RNase A/T1 Mix, Thermo ScientificTM, Catalog# EN0551) at 1 µg/mL for 30 min at 37°C or DNase (DNase I, Thermo ScientificTM, Catalog# EN0521) 1 U/mL for 30 min at 37°C. Following treatments, all samples underwent ultracentrifugation at 367,000 g for 16 h to isolate supermere pellets. The resulting pellets were resuspended in 200 µL of PBS and injected into mice via tail vein injection. After 24 h, the mice were euthanized, and organs were harvested for analysis, as described above. For EV distribution in normal and tumor brain, each group includes three mice, and for each mouse, five frozen brain sections were used, resulting in 15 data points per group. The negative control group consists of tumor‐bearing (GL261) mice injected with HEK‐293 supermere that were not labeled with NIR dye. This group includes one mouse with five brain sections, yielding five data points.

### 2.2 Immunofluorescence

Mice (CD‐1) were injected via tail vein with NIR‐conjugated supermeres, and 48 h later were perfused with 20 mL of PBS twice, followed by perfusion with 20 mL of freshly made 4% paraformaldehyde (PFA) in PBS. Brains were carefully collected, immersed in 4% PFA overnight at 4°C, and subsequently transferred to a 30% sucrose solution for dehydration for 48 h then embedded in Tissue‐Tek O.C.T. Compound (Sakura Finetek) and sectioned into 10 µm‐thick slices using a cryostat. The frozen sections were fixed in pre‐cooled acetone at −20°C for 10 min, followed by three washes with PBS, permeabilization was performed using 0.3% Triton X‐100 in PBS for 20 min at 4°C, sections were then blocked with 5% bovine serum albumin (BSA) in PBS for 1 h at 4°C to minimize non‐specific binding. Primary antibodies were applied to the sections and incubated overnight at 4°C in a humidified chamber. The following day, sections were washed three times with PBS and incubated with fluorescently labeled secondary antibodies at room temperature for 1 h, protected from light. After washing of secondary antibody staining, sections were mounted using a DAPI‐containing antifade mounting medium and covered with a coverslip. The slides were allowed to dry for 1 h at RT. The stained sections were imaged using a confocal microscope, and data were analyzed using ZEN Black and ZEN Blue software (Zeiss). The antibodies used are as follows: primer antibodies: anti‐NeuN (1:200, Cell Signaling #94403), anti‐MAP2 (1:100, Cell Signaling #8707), anti‐IBA1 (1:50, Cell Signaling #20825), anti‐MBP (1:100, Cell Signaling #78896), anti‐GFAP (1:400, Cell Signaling ##80788). Secondary antibodies: Anti‐mouse (1:1000, Alexa Fluor 488 Conjugate, #4408), Anti‐rabbit (1:1000, Alexa Fluor 594 Conjugate, #8889). For lectin immunofluorescence, mice received an intravenous injection of fluorescently labeled leptin (Invitrogen Lycopersicon Esculentum (Tomato) Lectin (LEL, TL), DyLight 488, catalog# L32470) via the tail vein at a dose of 5 mg/kg 30 min prior to euthanasia.

### 2.3 GBM PDX Cell Cultures

EGFRvIII‐positive PDX cells GBM6, GBM39 and GBM59 (Vaubel et al. 2020) were maintained in DMEM supplemented with 10% EV‐cleared Fetal Bovine Serum (FBS) (Atlanta Biologics) and penicillin‐streptomycin. Prior to harvest conditioned media for downstream analyses, cells were cultured in phenol‐red‐free FBS‐free DMEM supplemented with penicillin‐streptomycin for 24 h. The conditioned media was then depleted of whole cells (live and dead) and cell debris by centrifugation at 400 g for 10 min at 4°C and 2000 g for 20 min at 4°C.

EVs and NVEPs were isolated according to published protocols (Zhang et al. 2021, 2023) using a Beckman Optima XL‐90 ultracentrifuge equipped with SW28, SW41 Ti, SW32 Ti and SW55 Ti rotors (Beckman Coulter). To isolate 15k EVs, the conditioned medium was centrifuged at 15,000 g for 60 min at 4°C to pellet 15k EVs. The resulting pellet was resuspended in PBS and subjected to a second centrifugation at 15,000 g for 60 min at 4°C. To isolate 167k EVs, the 15k EV‐depleted supernatant was filtered through a 0.22 µm polyethersulfone filter (Nalgene) to minimize contamination from larger vesicles. The filtrate was concentrated using a centrifugal concentrator with a 100,000 molecular weight cutoff (Millipore). The concentrated sample was centrifuged at 167,000 g for 4 h at 4°C using an SW32 Ti swinging bucket rotor to pellet 167k EVs. The 167k EV pellet was resuspended in PBS containing 25 mM HEPES (pH 7.2) and further purified by washing through an additional centrifugation step at 167,000 g for 4 h under the same conditions. To isolate exomeres, the supernatant obtained from the final centrifugation step of 167k EV isolation was subjected to ultracentrifugation at 167,000 g for 16 h at 4°C. The resulting exomere pellet was resuspended in PBS containing 25 mM HEPES (pH 7.2) and washed via a second ultracentrifugation step at 167,000 g for 16 h. To isolate supermeres, the supernatant obtained after exomere isolation was further ultracentrifuged at 367,000 g for 16 h at 4°C using an SW55 Ti rotor. The resulting pellet, enriched in supermeres, was resuspended in PBS containing 25 mM HEPES (pH 7.2) and washed via a second ultracentrifugation step at 367,000 g for 16 h. All four types of EV and NVEP pellets were suspended in 200uL PBS containing 25 mM HEPES and stored at −80°C.

### 2.4 15k EVs and 167k EVs Flow Cytometry

Flow cytometry of EVs were performed using the vFC Vesicle Flow Cytometry EV Analysis Assay Kit (Cellarcus Biosciences, catalog #CBS4H‐5PE). 15k EVs and 167k EVs, derived from GBM cells, were diluted to a concentration of approximately 1 × 10⁶–1 × 10⁸ particles/µL in Staining Buffer. To assess EV size and EV surface cargo, GBM‐derived 15k EVs (50 ng per well) and 167k EVs (10 ng per well), along with vCAL NanoRainbow beads (included in the assay kit), were stained with vFRed Membrane Stain and the directly conjugated primary antibody antibodies, the staining reaction was carried out in the dark at room temperature (RT) for 60 min. Following staining, the reaction mixture was diluted 1:1000 in Staining Buffer performed using a CytoFLEX flow cytometer (Beckman Coulter) with a sample acquisition speed of 60 µL/min for 120 s per run. Data were collected and analyzed to determine EV size by De Novo Software. The primary antibodies used as directly conjugated antibodies were PE anti‐human EGFR Antibody (1:100 Biolegend Cat #352904), anti‐CD9 (1:50 Biolegend Cat #312106), anti‐CD63, (1:50 Biolegend Cat #353004), anti‐CD81 (1:50 Biolegend Cat #349506).

### 2.5 EV Size and Concentration Measurement by NTA

For quantitation of vesicle concentration and size distribution, cells were seeded in triplicate, grown to 80% confluency, washed twice with PBS, and grown overnight in phenol red‐free DMEM with 0.1% FBS. EVs were then harvested in fresh phenol red‐free DMEM with 0.1% FBS for 24 h. Conditioned media were cleared of live cells and cellular debris by centrifugation at 300 and 2000 g, respectively. The cleared media were imaged undiluted using a Nanosight LM10 (Malvern Instruments, Malvern, UK) with camera level = 15 and screen gain = 1.0. The range of particles per frame was between 10 and 100 particles per frame. Video recordings were analyzed using the NTA 3.1 software with screen gain = 3.0 essentially, as previously described (Gyuris et al. 2019). Each conditioned media sample was imaged three times and averaged. Following media collection, the cells were counted to give the denominator for the vesicles/mL/cells calculation. Each well was paired for cells and EVs and the corresponding value was averaged over the biological triplicate to yield the vesicle quantitation for each cell line.

### 2.6 Transmission Electron Microscopy

Freshly glow‐discharged formvar and carbon coated nickel 200 mesh grids (Electron Microscopy Sciences, Hatfield, PA) were floated on 10 mL drops of sample (ranging between 90 and 400 ng/mL of protein) to adsorb for 5 min before wicking the grid dry by touching the edge with filter paper. The grids were then floated on drops of 2% aqueous uranyl acetate (Electron Microscopy Sciences, Hatfield, PA) for 1 min and then wicked dry with filter paper and allowed to dry completely before imaging in a JEOL JEM‐1400 TEM (JEOL, Peabody, MA) at 120Kv equipped with Gatan Orius SC1000 digital CCD cameras (Gatan, Pleasanton, CA). Images were taken at the center and four quadrants of the grid. Size (diameter) measurements and quantification of EVs and ENPs was achieved using ImageJ.

### 2.7 Iodixanol Density Gradient Fractionation

Discontinuous step (12%–36%) gradients of iodixanol (OptiPrep) density media (Sigma‐Aldrich, St Louis, MO, USA) was prepared in ice‐cold PBS immediately before use. Pellets of 15k EVs and 167k EVs were resuspended in ice‐cold PBS and mixed with ice‐cold iodixanol/PBS to a final 36% iodixanol solution, which was added to the bottom of a centrifugation tube and solutions of descending concentrations of iodixanol/PBS were top‐layered individually to complete the gradient. Parallel gradients without sample were generated to determine fraction densities. The 12%–36% gradients were ultracentrifuged at 120,000 g for 15 h at 4oC using a SW41 TI Swinging Bucket rotor (k factor of 124, Beckman Coulter). Twelve individual fractions of 1 mL were collected from the top of the gradient. From the duplicate gradient, fraction densities were measured using a refractometer. For immunoblotting and silver stain SDS‐PAGE, each individual 1 mL fraction was transferred to new ultracentrifugation tubes, diluted 12‐fold in PBS and subjected to ultracentrifugation at 120,000 g for 4 h at 4°C using a SW41 TI swinging bucket rotor. The resulting pellets were lysed in RIPA cell lysis buffer on ice.

### 2.8 Proteomics

Samples for proteomic analysis were prepared as follows: 250 µg of proteins were extracted from the indicated fractions by lysing in 250 µL of 8 M urea, 200 mM EPPS pH 8.5 complemented with protease inhibitors (Pierce A32953) and lysed using a syringe (10 strokes with 1.5 inch, 21G needle) to shear the DNA. Samples were reduced with 5 mM of neutralized TCEP (Pierce 77720) for 15 min at room temperature and alkylated with 10 mM of iodoacetamide for 30 min in the dark at room temperature, followed by quenching with 5 mM DTT for 15 min at room temperature. 100 µg of protein was then extracted using methanol‐chloroform precipitations. Briefly, 400 uL of 100% methanol was added to the samples (100 µg in ∼100 µL) and vortexed for 5 s followed by the addition of 100 µL of 100% chloroform and vortexed for 5 s. 300 µL of water was added and samples were vortexed for 5 s and then centrifuged for 1 min at 14,000 g. The aqueous and organic phases were removed, carefully leaving a protein interphase in the tube. Samples were washed with 400 µL 100% methanol and centrifuged at 21,000 g for 2 min at room temperature, supernatant discarded and the pellet not allowed to dry completely. Samples were resuspended in 100 uL of 200 mM EPPS (pH 8.5).

For protein digestion and preparation of mass spectrometry analysis, the samples were resuspended in 100 µL of 100 mM EPPS, pH 8.5 and digested at 37°C with trypsin at a 100:1 protein‐to‐protease ratio overnight. The samples were desalted via StageTip (Rappsilber et al. 2007), dried via vacuum centrifugation, and reconstituted in 5% acetonitrile, 5% formic acid for LC‐MS/MS processing. Mass spectrometry data were collected using either: (1) an Exploris 480 mass spectrometer (Thermo Fisher Scientific, San Jose, CA) coupled with a Proxeon 1200 Liquid Chromatograph (Thermo Fisher Scientific) in DDA (data dependent acquisition) mode. Peptides were separated on a 100 µm inner diameter microcapillary column packed with approximately 35 cm of Accucore C18 resin (2.6 µm, 150 Å, Thermo Fisher Scientific). We loaded approximately 1 µg onto the column. Peptides were separated using a 75 min gradient of 3%–22% acetonitrile in 0.125% formic acid with a flow rate of 350 nL/min. The scan sequence began with an Orbitrap MS1 spectrum with the following parameters: resolution 120,000, scan range 350−1200 Th, automatic gain control (AGC) target: 300%, maximum injection time: 25 ms, RF lens setting :50%, and centroid spectrum data type. We selected the top twenty precursors for MS2 analysis which consisted of HCD high‐energy collision dissociation with the following parameters: resolution 30,000, AGC was set at “standard”, maximum injection time: 60 ms, isolation window: 1.2 Th, normalized collision energy (NCE) 28 and centroid spectrum data type. In addition, unassigned and singly charged species were excluded from MS2 analysis and dynamic exclusion was set to 60 s. (2) an Orbitrap Fusion Lumos instrument coupled to a Proxeon NanoLC‐1200 UHPLC. The 100 µm capillary column was packed with approximately 35 cm of Accucore 150 resin (2.6 µm, 150Å; ThermoFisher Scientific) at a flow rate of 360 nL/min. We loaded approximately 1 µg onto the column. Data were acquired for 70 min per sample. Peptides were separated using a 75 min gradient of 3%–22% acetonitrile in 0.125% formic acid with a flow rate of 350 nL/min. The scan sequence began with an MS1 spectrum (Orbitrap analysis, resolution 60,000, 400–1400 Th, automatic gain control (AGC) target 100%, maximum injection time 50 ms). The hrMS2 stage consisted of fragmentation by higher energy collisional dissociation (HCD, normalized collision energy 30%) and analysis using the Orbitrap (AGC 200%, maximum injection time 22 ms, isolation window 1.3 Th, resolution 15K). Data were acquired using the FAIMSpro interface the dispersion voltage (DV) set to 5000 V, the compensation voltages (CVs) were set at −40 V, −60 V and −80 V, and the TopSpeed parameter was set at 1 s per CV.

#### 2.8.1 Mass Spectrometric Data Analysis

MS spectra were converted to mzXML using a modified version of ReAdW.exe. Database searching included all entries from the human UniProt database (downloaded September 2020), which was concatenated with a reverse database composed of all protein sequences in reversed order. Searches were performed using a 50‐ppm precursor ion tolerance and the product ion tolerance was set to 0.03 Th (Beausoleil et al. 2006). Up to two missed cleavages were allowed, charge states between 2 and 6 were permitted, and peptide length was limited to a range between 7 and 60 residues. Oxidation of methionine residues (+15.9949 Da) was set as a variable modification. Peptide spectral matches (PSMs) were altered to a 1% FDR (Elias and Gygi 2010; Elias and Gygi 2007). PSM filtering was performed using a linear discriminant analysis, as described previously (Huttlin et al. 2010), while considering the following parameters: XCorr, ΔCn, missed cleavages, peptide length, charge state and precursor mass accuracy. Peptide‐spectral matches were collapsed to a 1% FDR and then further collapsed to a final protein‐level FDR of 1%. Furthermore, protein assembly was guided by principles of parsimony to produce the smallest set of proteins necessary to account for all observed peptides. For quantification and downstream data analysis, spectral counts were extracted and the subsequently converted to Normalized spectral counts abundance factors, as outlined previously (Paoletti et al. 2006). For the analysis of proteomics raw data, the normalized spectral abundance factor (NSAF) data were used given its heightened precision (Neilson et al. 2013). Missing proteins in more than three sample replicates were filtered out and quantile normalization was applied to the samples. Proteomics data containing processed ms data with protein identities, values used for quantitation, *q* values/adj *p* values and FC values are included in Tables S5–S7. To identify proteins from EVs/NVEPs enriched in Biological Process (BP), MF and Cellular Component (CC), we utilized the Database for Annotation, Visualization and Integrated Discovery (DAVID) v7.0 (Huang da et al. 2009, Huang da et al. 2009) with Gene Ontology. All terms with a *p* value (Benjamini or Benjamini–Hochberg adjusted) < 0.05 were considered significant and ranked by the number of proteins identified in the group.

### 2.9 Western Blotting

Protein from the various EV and NVEP preparations were isolated in RIPA lysis buffer and quantitated by BCA. Equivalent amounts of protein from each fraction were loaded on SDS‐PAGE gels and analyzed for equal loading by silver staining. Corrected equal amounts of protein were run and examined by western blotting. The antibodies used are as follows: primer antibodies: anti‐EGFR, (1:1000, CST, #4267), anti‐CD9,(1:1000, CST, #13403), anti‐CD63, (1:1000, CST, #52090), anti‐CD81, (1:1000, CST, # 52892), β‐Actin, (1:1000, CST, #3700), anti‐Calnexin, (1:1000, CST, #2433), anti‐Annexin A2, (1:1000, CST, #8235), anti‐Cytochrome C, (1:500, CST, #11940). Secondary antibodies from LI‐COR, (1:10000, IRDye 680RD Goat anti‐Mouse, #926‐68070, IRDye 800CW Goat anti‐Rabbit, # 926–32210).

### 2.10 Transcriptomics

For bulk RNAseq, total RNA was isolated from EVs and NVEPs using Qiagen microRNA isolation kit, as recommended by manufacturer. Total cellular RNA was isolated from the corresponding source PDX cultures and analyzed in parallel. The concentrations of cellular and EV/NVEPs RNA were determined by spectrophotometer (NanoDrop 2000) and Quant‐iT RiboGreen RNA Assay Kit (Thermo Fisher Scientific), respectively. The RNA quality was examined using Agilent 2100 Bioanalyzer (Agilent, CA) and the RNA Integrity Numbers (RIN) were estimated. Total RNA, either 40–200 ng of EV/NVEPs RNA, or 2 µg of cellular RNA, were rRNA‐depleted using the Ribo‐Zero rRNA Removal Kits (Illumina, CA). One quarter of the rRNA‐depleted RNA was fragmented to 100–500 nts using the 5× First‐Strand Buffer (Clontech, CA), and utilized for the bulk RNA library construction by SMARTer Stranded RNA‐Seq Kit (Clontech). The quality of libraries was examined using the Agilent DNA 1000 kit at the Agilent 2100 Bioanalyzer instrument, and cDNA quantified by qRT‐PCR. The libraries were sequenced on HiSeq 2000 (Illumina) with single‐read 75 cycles at the Dana Farber Harvard Medical School Sequencing Core facilities. RNA‐seq raw reads were reversely‐stranded paired‐end reads. The reads were trimmed for adapters and polyX tails, then filtered by sequencing Phred quality (≥ Q15) using fastp (Chen et al. 2018). The adapter‐trimmed reads were aligned to the genome using STAR aligner with the two‐pass option. Reads were mapped across the genome to identify novel splice junctions in the first‐pass. These new annotations were then incorporated into the reference indexes and reads were re‐aligned with this new reference in the second pass. To estimate the gene expression from the genome alignments, RSEM, a tool for accurate quantification of gene and isoform expression from RNA‐Seq data (Li and Dewey 2011) was used. The reads were then aligned to the human transcriptome (Ensembl version 104) using kallisto (Bray et al. 2016) and converted transcript counts to gene counts using tximport (Soneson et al. 2015). The proportions of the gene biotypes of the mapped gene counts were derived from transcriptome alignment. Note that the rRNA species were excluded, and Figures 5B and S5C show only the top 12 most abundant species, and all the remaining species are collapsed. To filter out low expressing genes, genes that have counts per million (CPM) more than 0.46 in at least four samples were retained. There were 43,060 genes after filtering. To use linear models in the following analysis, Voom transformation (Law et al. 2014) was performed to transform counts into logCPM, where 𝑙𝑜𝑔𝐶𝑃𝑀 = 𝑙𝑜𝑔2(106×count/library size). Voom transformation estimated the mean‐variance relationship and used it to compute appropriate observation‐level weights so that more read depth gave more weights. To extract differentially expressed genes, we used limma, an R package that powers differential expression analyses (Ritchie et al. 2015), and performed linear modeling and moderated *t*‐tests to detect genes that were differentially expressed between groups, with the SVs as covariates. To discover genes that are differentially expressed among the five groups, we used the R package limma (Ritchie et al. 2015) to perform linear modeling and moderated *F*‐tests, with the adjustment of the SVs. Significant genes (*F*‐test FDR < 0.01) were subsequently selected. Hierarchical cluster analysis based on the Euclidean distance of these selected genes was performed and gene clusters in the hierarchical dendrogram using a variable cut height approach (Langfelder et al. 2008) were detected.

For small RNA‐seq, high‐throughput small RNA sequencing (sRNA‐seq) was completed using the Illumina short‐read platform. Total RNA was isolated from cells, EVs and NVEPs using miRNAEasy Isolation Kits (Qiagen). sRNA libraries were generated using the NEXTFlex Small RNA Library Preparation Kits v3 with UDIs (Revvity), using the following modifications: (1) 3′‐ and 5′‐adaptors were diluted 1:8, (2) 3′‐adaptor ligation was performed overnight in multiple steps –25°C for 2 h, 20°C for 4 h and 16°C overnight, (3) following cDNA synthesis and before barcoding PCR, modified step F protocol was used and (4) PCR amplification of 20 cycles. Individual libraries were size‐selected (136–200 bp) using PippinPrep pre‐cast cassettes (Sage Sciences) and quantified on a Qubit Fluorometer. Paired‐end sequencing (PE‐150) of equimolar multiplexed libraries were completed using the NovaSeq6000 (Illumina) at the VANTAGE DNA Sequencing core (Vanderbilt University, Nashville, TN). Down‐stream analyses were completed using the TIGER data analysis pipeline (https://github.com/shengqh/TIGER)). Cutadapt (v1.16) was used for 3′ adaptor trimming and reads with >16 nts were used for alignments (hg19 genome), with additional rRNA and tRNA reference sequences, by Bowtie1 (v1.1.2) allowing only one mismatch (1 MM). Reads <20 nts that failed to be annotated as sRNA without perfect alignment to human genome were discarded. For the analysis of class composition in both small and long RNA libraries, the total abundance of corrected non‐rRNA was used for normalization between the samples. The hierarchical clustering analyses were performed on log2‐transformed, normalized read counts using the Limma R package. Venn diagrams were generated using identified differentially localized gene names input into the Venn function in the gplots R package and further annotated in Adobe Illustrator.

### 2.11 Lipidomics

Liquid chromatography tandem mass spectrometry (LC‐MS/MS) was used to identify and quantify non‐covalently bound lipids extracted from EVs (15k × *g* and 167k × *g* fractions) in the Mass Spectrometry Core at Vanderbilt University medical Center. Lipids were extracted by Bligh–Dyer lipid isolation method and analyzed using a Vanquish Horizon UPLC system (Thermo) system prior to injection into a QExactive hybrid quadrupole/Orbitrap high‐resolution mass spectrometer (Thermo). MS Dial v4.9(1, 2) were used for down‐stream analyses. Equi‐volume pooling of all samples were used as a quality control (QC) sample. Blanks, solvent negative control and aqueous internal standards (AIS; Equisplash lipidomix, Avanti) were also included. Lipid species with (a) relative standard deviation (RSD) >20% for QC sample, (b) blank/QC values >10% and (c) nomenclature issues (unknowns, w/0 ms2 and RIKEN) were removed. Data were normalized using the LOWESS method. For the exomeres and supermeres analysis, lipids were isolated from fractions using a liquid–liquid methyl‐tert‐butyl ether (MTBE) extraction method (Matyash et al. 2008) designed for phase separation of non‐polar compounds and analyzed on a LC‐MS/MS platform developed at the Mass Spectrometry (Proteomics/Metabolomics) Core at Beth Israel Deaconess Medical Center was used (Breitkopf et al. 2017). This high‐resolution lipidomics program uses positive/negative ion switching with C18 reversed‐phase chromatography in data dependent analysis (DDA) mode on a QExactive Plus Orbitrap mass spectrometer (Breitkopf et al. 2017). Individual intact lipid molecules were identified using LipidSearch 4.1 software (Yamada et al. 2013).

### 2.12 Digital Flow Cytometry

One hundred and thirty microliters of 5 × 10^9^ vesicles/mL of GBM 15k EV and 167k EV samples in DPBS were centrifuged at 12,000 RCF for 2 min through a Spin‐X Centrifuge Tube Filter with 0.45 µm pores on a cellulose acetate membrane (Corning, New York, Costar Spin‐X, Cat#8162). Next, 2.0 µL of 14 nM of each antibody, Anti‐CD9‐PE (Biolegend, California, Catalog: 31205, Lot: B329604), Anti‐CD81‐PE/Cy7 (Biolegend, California, Catalog: 349511, Lot: B348509), Anti‐CD63‐Alexa488 (Biolegend, California, Catalog: 353037, Lot: B336115) and Anti‐EGFR‐Alexa647 (Biolegend, California, Catalog: 352917, Lot: B369099), were added to the sample and then incubated at RT in the dark for 1 h. Then, 1.3 uL of 10 µM di‐8‐ANEPPS (ThermoFisher, Massachusetts, Catalog: D3167, Lot: 2438355) was added and incubated at RT in the dark for 1 h. The sample was then purified through an SEC column with 30 nm pore size (Exo‐spin mini, CELL guidance systems, Missouri, Catalog: EX03). Then, 2 µL of 0.05% BSA (UltraPure BSA (50 mg/mL), ThermoFisher, Massachusetts, Catalog: AM2618) was then added to 98 µL of eluate and 10 µL of this mixture was loaded onto the microchip and placed on dFC (Kim et al. 2024; Andronico et al. 2021; Jiang et al. 2021; Jung et al. 2018) for analysis.

### 2.13 Cellular Uptake Assays

HMC3 cells were seeded in six‐well plates and cultured until cell confluence reached approximately 90%. At this point, supermere particles labeled with IRDye 800 were introduced to the cells to assess uptake. Specifically, 50 µg of IRDye 800‐labeled supermere in 200 µL of PBS was added to each well. Following a 6‐h incubation at 37°C, the cells were washed three times with PBS to remove any unbound supermere particles. The treated cells were then harvested, and the uptake of IRDye 800‐labeled supermere was quantified by flow cytometry. The percentage of cells positive for IRDye 800 fluorescence was determined, providing a measure of supermere internalization.

### 2.14 qRT‐PCR

HMC3 cells were treated with lipopolysaccharide (LPS) at a final concentration of 50 ng/mL, along with supermere particles from GBM6, GBM39 and GBM59 and HEK‐293 and GL261 cells at concentrations of 50 µg/mL each. Following a 24‐h incubation at 37°C, the cells were washed three times with PBS to remove any unbound supermere particles. RNA was then extracted from the treated cells using the QIAwave DNA/RNA Mini Kit (QIAGEN, Catalog# 80504), following the manufacturer's protocol. Complementary DNA (cDNA) synthesis was performed using the HiScript IV RT SuperMix for qPCR (+gDNA wiper) (Catalog# R423‐01), and qPCR was carried out using the Taq Pro Universal SYBR qPCR Master Mix (Catalog# Q712‐02), according to the manufacturer's guidelines. The primer sequences of the target genes are reported in Table S8. Gene expression was quantified using a standard qRT‐PCR protocol, and relative expression levels were calculated using the comparative Ct method. All assays were performed in triplicate, and appropriate controls were included in each experiment. LPS levels in supermeres were assessed using an LPS assay kit (ThermoFisher, #A39552) according to the manufacturer's protocol.

### 2.15 Statistics

Values are given as mean ± SEM or SD as indicated. Numbers of experimental replicates are given in the figure legends. When two groups were compared, significance was determined using an unpaired two‐tail *t*‐test. A *p* value < 0.05 is considered as statistical significance.

## 3 Results

### 3.1 GBM PDX Secrete EVs of Different Densities

**FIGURE 1:**
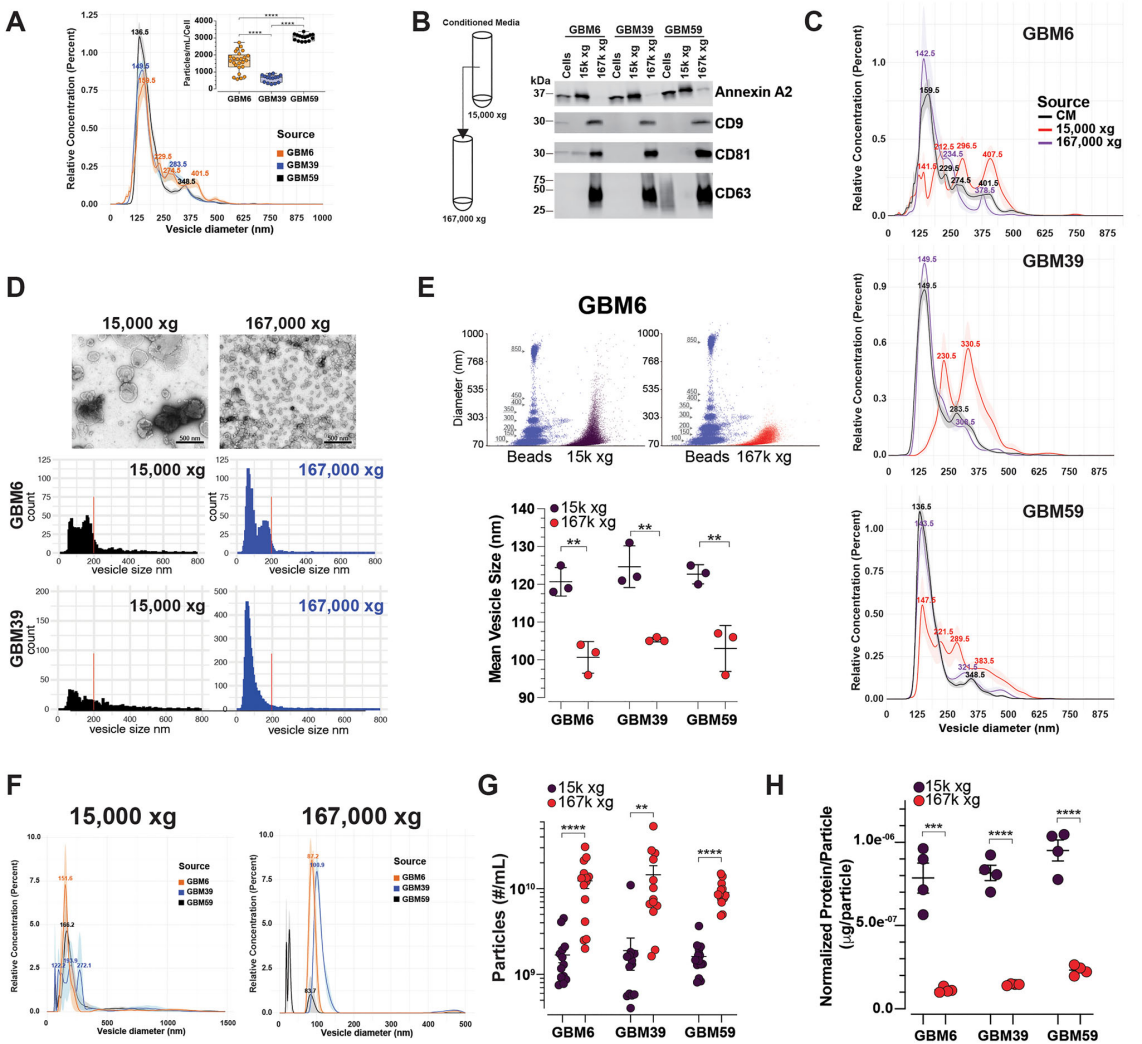
**Differential ultracentrifugation sediments EVs of similar diameter. (A)** Representative NTA (Nanosight) trace plot of conditioned media from the indicated GBM PDX cell cultures. Inset; rate of EV production as determined by concentration of particles per mL normalized to number of cells. Data are presented as the mean ± SEM of biologically independent replicates (GBM6 *n* = 6 in technical *n* = 4, GBM39 and GBM59 *n* = 3 in technical *n* = 5), unpaired *t* test, two‐tailed, **** *p* < 0.0001. **(** **B)** Graphical representation of the differential UC approach and representative western blot of the indicated EV markers. **(** **C)** Representative NTA (Nanosight) trace plot of EVs isolated using differential UC (15k × *g* and 167k × *g*) for the indicated GBM PDX cell cultures. **(** **D)** Representative photomicrographs of TEM and quantitation of EV sizes (binned in 10 nm increments) from differential UC (15k × *g* and 167k × *g*) of the indicated GBM PDX cell cultures. **(** **E)** Representative flow cytometry plot and quantitation of vesicle size from differential UC (15k × *g* and 167k × *g*) isolated EVs from GBM‐6 PDX cell culture. Bead marker sizes are indicated in nm. Data are presented as the mean ± SD of biologically independent replicates, *n* = 3, unpaired *t* test, two‐tailed, **p < 0.01. **(** **F)** Dynamic light scattering (DLS) profiles of 15k × *g* and 167k × *g* EV preparations of the indicated human GBM PDX cultures. **(** **G)** Nanoflow vesicle flow cytometry applied on CM‐isolated 15k × *g* and 167k × *g* preparations. Data are presented as the mean ± SEM of biologically independent replicates, unpaired *t* test, two‐tailed, **p < 0.01, **** *p* < 0.0001. **(** **H)** Protein amounts normalized to vesicle numbers. Data are presented as the mean ± SEM of biologically independent replicates (*n* = 4), unpaired *t* test, two‐tailed, ***p < 0.001, **** *p* < 0.0001.

EGFR^vIII^ is a potent oncogene that is observed in approximately 35% of GBM patients. To study EVs produced by EGFR^vIII^ positive GBMs, we first analyzed the size distribution and concentrations of EVs from conditioned media isolated from human GBM PDX lines GBM6, GBM39 and GBM59 (Vaubel et al. 2020) that express measurable levels of EGFR^vIII^ using nanoparticle tracking analysis (NTA) (Figure 1A). The peak sizes of the majority of EVs produced ranged between 136.5 and 159.5 nm in diameter (Figure 1A) with particle production (as measured using concentrations within a 24 h collection period) differing significantly between all three lines ranging between 700 and 3100 particles/mL/cell/24 h (Figure 1A inset). This production was independent of EGFR expression and activation levels (Figure S1A); however, it inversely correlated with the PDX cell culture growth rates (Figure S1B).

Most vesicles had a size range of 136.5–159.5 nm, but there were small numbers of vesicles larger than 200 nm. To determine whether the larger EVs are ontologically distinct from the smaller EVs, we used differential centrifugation to isolate vesicles. We centrifuged conditioned media from the GBM PDX cells at 15,000 × *g* and 167,000 × *g* and designated these vesicles as 15k and 167k EVs, respectively, according to MISEV 2023 guidelines (Welsh et al. 2024) (Figure 1B). 167k EVs were enriched for the canonical tetraspanin markers CD9, CD63 and CD81, whereas 15k EVs were enriched for Annexin A2 by western blot (Figure 1B). Surprisingly, NTA of 15k and 167k EVs prepared from the GBM PDX cell lines by ultracentrifugation pelleting revealed considerable amounts of approximately 150 nm diameter particles in the slower speed UC 15k EV preparations in addition to larger particles (Figure 1C). On the other hand, the 167k EV pellets contain mostly particles of peak diameters ranging between 142.5 and 149.5 nm (Figure 1C).

To validate the size distribution of 15k and 167k EVs, we performed Transmission Electron Microscopy (TEM) and measured vesicle diameters. Low‐speed ultracentrifugation (15,000 × *g*) can pellet vesicles of various sizes (Jeppesen et al. 2019). To obtain a better separation of vesicles within the 15k EV pellet, we used iodixanol density gradient ultracentrifugation (DGUC) (Jeppesen et al. 2019), which was then followed by TEM on the various fractions obtained (Figure S1C–G). We observed that DGUC did not separate smaller vesicles (<200 nm) from larger vesicles (>200 nm) in both 15,000 × *g* and 167,000 × *g* UC pellets and that the majority of EVs are composed of vesicles <200 nm diameter, similar to NTA and TEM. Furthermore, we used vesicle flow cytometry on 15k and 167k EV preparations and noticed that although 15k EV preparations contain larger (>200 nm) vesicles, the major contributing vesicle species are smaller (<200 nm) (Figures 1E and S1H–J). Finally, we used dynamic light scattering to measure the diameter of vesicles from 15k and 167k EV preparations and found that the peak size of 15k EVs and 167k EVs ranged between 122.2–272.1 nm and 83.7–100.3 nm, respectively (Figure 1F). Using nanoflow cytometry (Kim et al. 2024), we determined that there are more 167k EVs than 15k EVs in CM. We observed 7.3 ± 1.0‐fold differences in the number of 167k EVs when compared to 15k EVs in GBM6, 7.7 ± 1.8‐fold in GBM39 and 5.6 ± 0.6‐fold in GBM59 (Figure 1G). Quantitating protein levels in 15k and 167k EVs revealed significantly higher amounts of proteins on a per vesicle basis in 15k EVs than 167k EVs, with 7.0 ± 0.3‐fold in GBM6, 5.6 ± 0.1‐fold in GBM39 and 4.1 ± 0.2‐fold in GBM59 (Figure 1H).

Using these orthogonal approaches, we demonstrated that low speed UC (15,000 × *g*) sedimented EVs are mostly composed of <200 nm diameter vesicles, similar to that of 167,000 × *g* pelleted EVs. We also found that the 167k EVs were present in higher concentrations than 15k EVs in conditioned media of GBM PDX cells. Surprisingly, we also observed higher levels of proteins in 15k EVs than 167k EVs on a per vesicle basis. Since centrifugal sedimentation of vesicles is related to their density, our observations raise the possibility that EVs that sediment at a lower UC speed in the 15k EV preparations have higher densities than the EVs pelleted at high‐speed UC.

### 3.2 GBM PDX‐Secreted 15k and 167k EVs Transport Divergent Lipidomes

**FIGURE 2:**
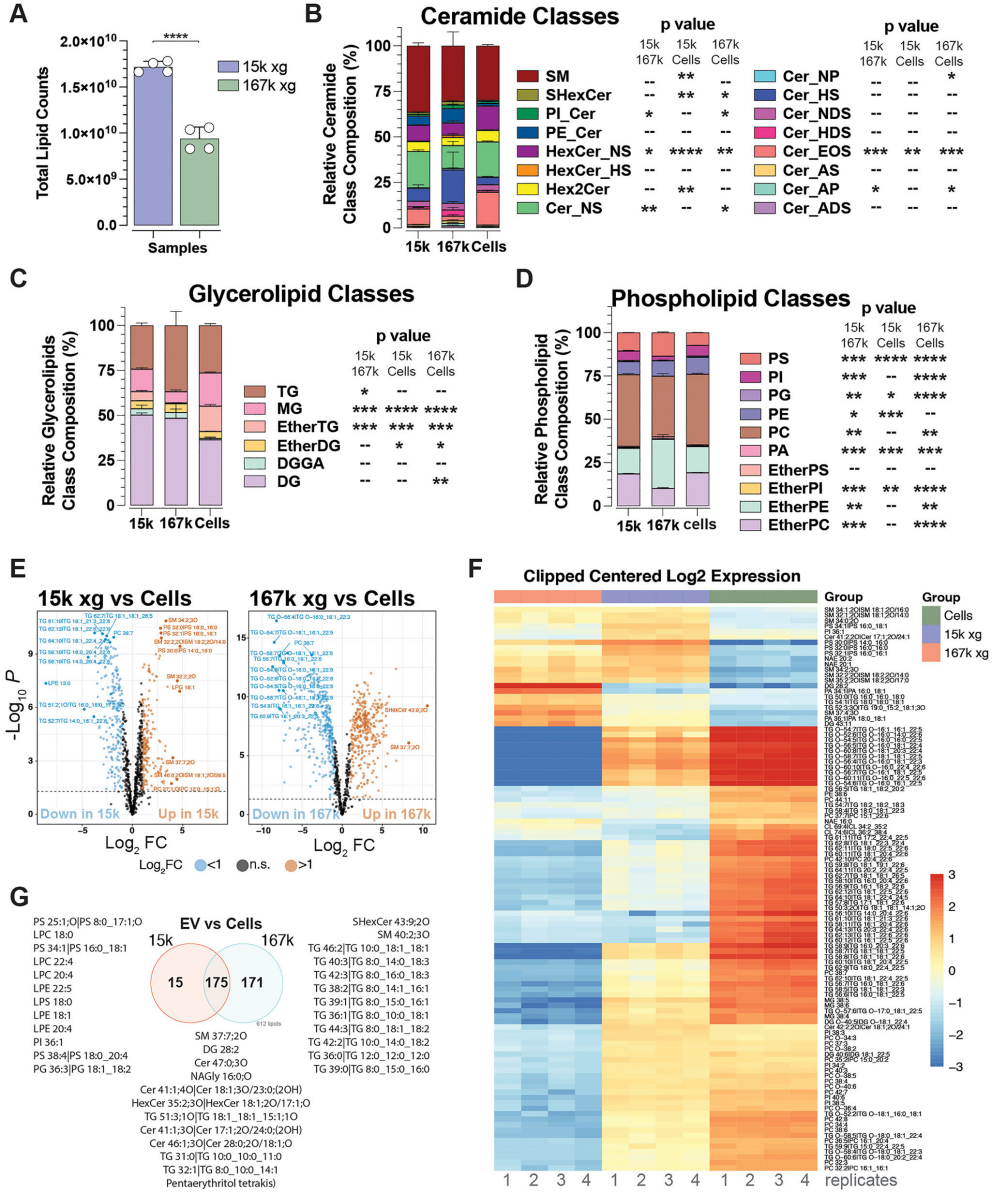
**Differential UC EVs have distinct lipid compositions. (A)** Total lipid counts of the 15k × *g* and 167k × *g* EV fractions isolated from GBM39. (**B–D)** Relative lipid subclasses composition (percentage) for ceramides (B), glycerolipids (C), and phospholipids (D). Data are presented as the mean ± SEM of biologically independent replicates (*n* = 4), unpaired *t* test, two‐tailed, *p < 0.05, **p < 0.01, ***p < 0.001 and ****p < 0.0001. (**E)** Volcano plots of total lipids from 15k × *g* and 167k × *g* EVs. (**F)** Heat map of top ranked lipids of each indicated fractions. (**G)** Venn diagram of enriched (Log2FC > 1) 15k × *g* and 167k × *g* EV lipids when compared to cellular levels. Indicated are the top (highest Log2FC down) 12 lipids for each segment.

EVs are bilayer membranous particles that carry soluble protein and nucleic acid cargos. To identify differences in phospholipids, and other lipid cargo between EVs classes, liquid chromatography tandem mass spectrometry (LC‐MS/MS) was performed on lipids isolated from UC‐purified 15k × g and 167k × g fractions derived from conditioned media of GBM39 cells and GBM39 cell extracts. We identified 973 molecular lipid species relative to internal standards, which categorized into eight groups and 60 subgroups (Table S1). Also, we observed a 1.83 ± 0.25‐fold increase in total lipid content in the 15k × *g* fraction relative to the 167k × *g* fraction (Figure 2A).

Furthermore, we observed that 15k and 167k EVs have relatively different lipid compositions when compared to their cell of origin (Figure S2A). Within ceramide lipid subclasses, we observed significant variations between EVs and cells in Ceramide EOS with a 7.28 ± 0.36‐fold decrease and a 2.09 ± 0.11‐fold decrease in 15k EVs and 167k EVs, respectively, when compared to cellular levels, respectively, and HexosylCeramide Non‐hydroxylated Sphingosine base (HexCer NS) with 1.95 ± 0.16‐fold and 1.50 ± 0.04‐fold decreases in 15k EVs and 167k EVs, respectively, when compared to cellular levels, respectively (Figure 2B). In the glycerolipid subclasses, we observed the most significant increases in DG in 15k and 167k EVs of 1.37 ± 0.04‐fold and 1.33 ± 0.17‐fold, respectively, when compared to cells, respectively (Figure 2C). In addition, there was a 2.75 ± 0.14‐fold reduction of Ether TG in 15k EVs compare to cells and a 38.6 ± 0.11‐fold reduction in 167k EVs when compared to cells (Figure 2C). Similarly, there was a 2.93 ± 0.17‐fold reduction of MG in 167k EVs and a 1.48 ± 0.08‐fold reduction in 15k EVs when compared to cells (Figure 2C). Within the phospholipid class, 167k EVs contained significantly more PS, PE and etherPE than 15k EVs, whereas levels of PI, PC and etherPC were significantly greater in 15k EVs than 167k EVs (Figure 2D). There were minimal differences amongst the most abundant lipid constituents between 15k, 167k EVs and cellular levels in Oxydized lipid, Lyso lipid and other lipid classes (Figure S2B–D).

At the single lipid level, when compared to cells, 15k EVs and 167k EVs had differences for several individual lipids (Figure 2E–G). For example, 167k EVs contained much higher levels of diacylglycerol (DG) and phosphatidic acid (PA) than 15k EVs and cells (Figure 2F). Moreover, several species of lysolipid classes were highly enriched in 15k EVs when compared to cells, whereas several triglycerol species appeared to be preferentially enriched in 167k EVs when compared to cells (Figure 2G). Overall, these results indicate that the lipid composition of 15k and 167k EVs differs from each other and their cell of origin, with 15k EVs resembling more cellular levels of lipids, whereas 167k EVs lipidome is similar to that of intracellular membrane sources (Llorente et al. 2013; Skotland et al. 2023). The higher levels of lipids contained in 15k EVs, as well as these compositional differences, likely contribute to their higher density than 167k EVs.

### 3.3 Proteomic Profiling of the 15k and 167k EVs

To better understand the ontology of EVs, we assessed the protein composition of donor cells and UC‐purified 15k and 167k EV fractions for GBM6, GBM39 and GBM59 PDXs using large scale liquid chromatography‐tandem mass spectrometry (LC‐MS/MS). We hypothesized that the selective loading of specific proteins into EVs is an active process rather than a passive one, with preferential cargo composition reflecting mechanisms tied to vesicle origin. To test this, we conducted comparative proteomic analyses of EVs and the cells from which they were produced.

**FIGURE 3:**
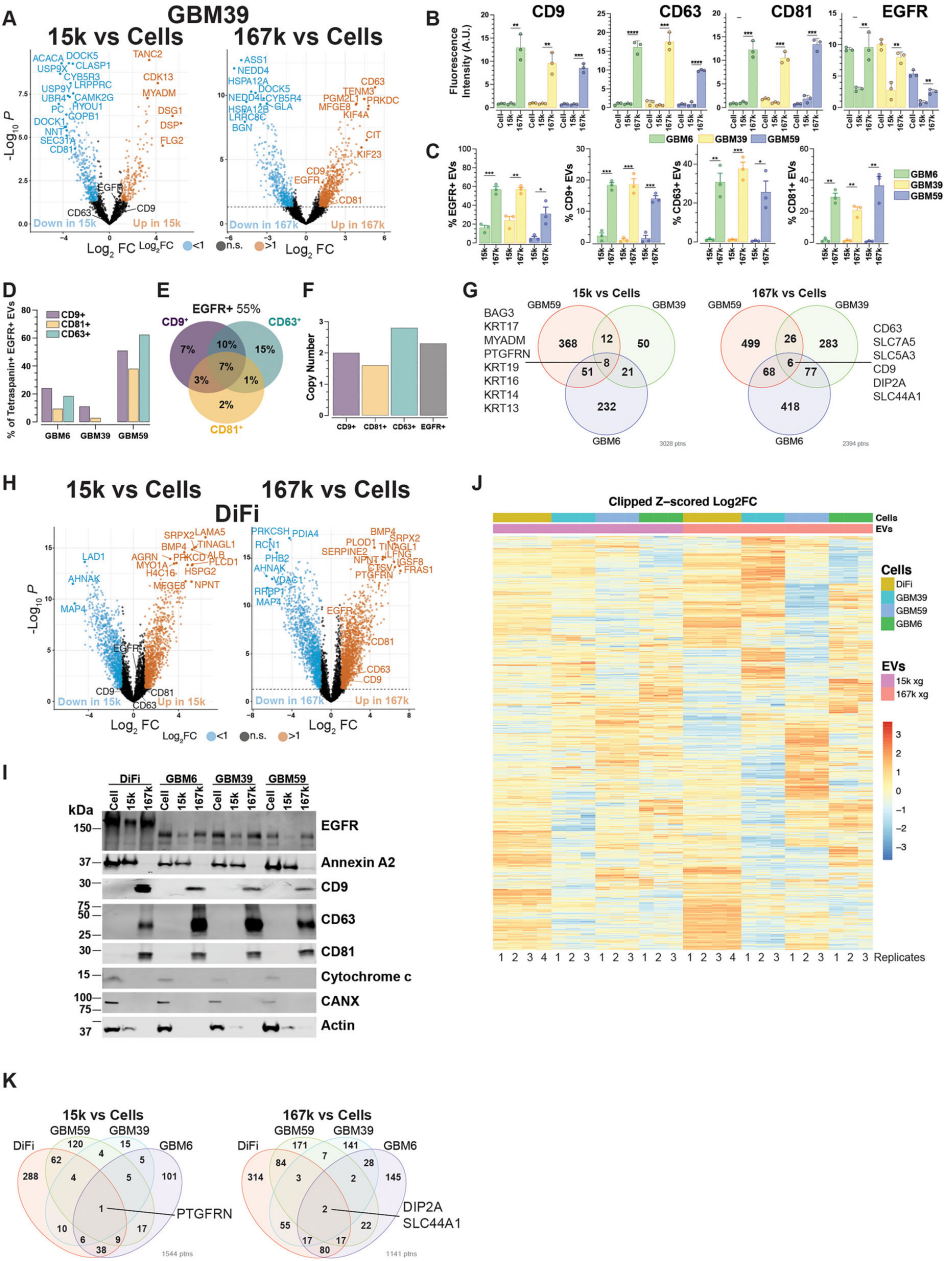
**Proteomes of 15k and 167k EVs have unique compositions. (A)** Volcano plots of proteins from GBM6 15k and 167k EV fractions versus cells. EGFR, CD9, CD63, and CD81 markers are highlighted. (**B)** Graphical representation of quantitation of vesicle nano‐flow cytometry of the indicated markers in cells, 15k and 167k EV fractions. Data are presented as the mean±SEM of biologically independent replicates (*n* = 3), unpaired *t* test, two‐tailed, **p < 0.01, ***p < 0.001 and ****p < 0.0001. (**C)** Single‐molecule vesicle flow cytometry for the indicated markers in 15k and 167k EVs isolated from GBM6, GBM39 and GBM59. Data are presented as the mean ± SEM of biologically independent replicates (*n* = 3), unpaired *t* test, two‐tailed, *p < 0.05, **p < 0.01, and ***p < 0.001. (**D)** Percentage of indicated GBM PDX cell‐derived 167k EV double positive for EGFR and the indicated tetraspanin markers among the entire population of EGFR‐positive EVs for the three GBM samples. (**E)** Venn diagram of percentages of GBM59‐derived 167k EVs positive for the indicated markers using digital flow cytometry. (**F)** Deconvolution of the signal intensity distributions for each indicated antibody marker representing copy number from digital flow cytometry from 167k EVs isolated from GBM59 PDX cells. (**G)** Venn diagram of protein species preferentially present (Log2FC >1) in GBM 15k and 167k EVs versus cells. Proteins that are common to all three GBM 15k and 167k EVs are indicated. (**H)** Volcano plots of proteins from DiFi cells 15k and 167k EV fractions versus cells. EGFR, CD9, CD63 and CD81 markers are highlighted. (**I)** Western blot of proteins isolated from 15k and 167k EV fractions and cells from GBM6, GBM39, GBM59 and DiFi cells for the indicated markers. (**J)** Heat map of Log2FC of proteins in 15k and 167k EV versus cells from DiFi, GBM6, GBM39 and GBM59 cells. Data are from biologically independent replicates (DiFi *n* = 4, GBMs *n* = 3). (**K)** Venn diagram of protein species preferentially present (Log2FC > 1) in DiFi and GBM 15k and 167k EVs versus cells. Proteins that are common to all four categories are indicated.

A total of 3772 proteins were detected in cells and 15k and 167k EVs from GBM6, GBM39 and GBM59 PDXs. The proteomic profiles of the 167k EV fractions were distinct from those of their respective 15k EVs and cells, with the latter two displaying a marked overlap (Figure S3A). The abundance of each protein in 15k EVs and 167k EVs was compared to cellular levels and those proteins displaying a Log_2_FC > 1 were considered preferentially enriched in EVs, some of which could represent EV‐specific markers (Figures 3A and S3B). EGFR and the canonical tetraspanin EV markers CD9, CD63 and CD81 were all enriched in the 167k EV fractions (Figures 3A and S3B).This higher abundance in 167k EVs was validated by vesicle flow cytometry (Kim et al. 2024), demonstrating a significant enrichment of these markers in 167k EVs when compared to 15k EVs and cells (Figures 3B and S3C,D). We observed a similar trend in the percentage of 167k EVs containing higher levels of EGFR, CD9, CD81 and CD63 than 15k EVs using single‐fluorophore digital flow cytometry of EVs labeled with the lipid membrane di‐8‐ANEPPS (Figure 3C) (Kim et al. 2024; Andronico et al. 2021; Jiang et al. 2021; Jung et al. 2018). Multi‐marker vesicle flow cytometry revealed that EGFR‐positive EVs were detected within populations of CD9+, or CD63+, or CD81+ EVs (Figure 3D) and, of the EGFR positive GBM39 167k EVs, 7% express all three tetrespanins, CD9, CD81 and CD63 (Figure 3E). Then, we evaluated the copy number of individual molecules for 167k EVs and determined that approximately two copies of CD9, CD81, CD63 and EGFR were present on each vesicle (Figure 3F).

Focusing on proteins with significant enrichment in EVs over cells, we identified proteins that are unique to either 15k EVs or 167k EVs as well as proteins that are observed in both (Figure S3E). By comparing 15k EV‐ and 167k EV‐enriched proteins from all three GBM PDX cultures, we identified eight proteins (BAG3, MYADM, KRT13, 14, 16, 17, 19 and PTGFRN) in 15k EVs and six proteins (CD63, CD9, DIP2A, SLC44A1, SLC5A3 and SLC7A5) in 167k EVs that are common amongst all three GBM PDXs (Figure 3G). Proteins common amongst all three GBM PDX lines from 167k EVs include known EV tetraspanin proteins, such as CD9 and CD63 in addition to DIP2A, a little characterized protein suspected to have function in axon guidance and synaptic plasticity (Zhang et al. 2023). In addition, solute carrier (SLC) proteins, which are membrane bound transport proteins responsible for moving a wide variety of solutes across cellular membranes, are also observed in all three GBM lines. On the other hand, proteins specific to 15k EVs from the GBM lines are involved in membrane dynamics and protein structure integrity (Figure 3D). For example, BAG3 is a co‐chaperone of HSP70 that regulates protein folding and degradation, MYADM is a lipid raft‐localized transmembrane protein with functions in membrane organization and cell signaling, and PTGFRN is an EV scaffold protein (Dooley et al. 2021).

Gene ontology (GO) analysis of proteins presents in at least two out of three GBM PDX lines suggested that the proteins enriched in the 15k EVs play a role in biological processes of skin and epidermis development as well as multivesicular body assembly (Figure S3F). Additionally, proteins in 15k EVs are part of GO Cellular Components (CC) that are associated with cell‐substrate junction, secretory granule membrane and ESCRTI complex and GO Molecular Functions (MF) involved in cadherin binding, glutamate receptor binding and structural constituent of cytoskeleton (Figure S3F). On the other hand, 167k EV proteins present in at least two out of three GBM PDX lines demonstrated GO Biological Processes (BP) of proteins localization to the plasma membrane, cell‐substrate adhesion and cytokinesis (Figure S3F). Furthermore, 167k EV proteins were also found to be involved in early endosome and basal plasma membrane CC and GTPase activator activity, GTP binding and protein tyrosine kinase activity (Figure S3F).

To determine whether these 15k EV‐ and 167k EV‐enriched proteins are specific to GBM, we isolated 15k and 167k EV fractions along with cell extracts from the colorectal cancer (CRC) cell line DiFi (Olive et al. 1993) and analyzed the DiFi‐secreted proteome by LC MS/MS. A total of 5973 DiFi proteins were identified in cells, 15k EVs and 167k EVs with PCA revealing that their proteomic profiles are distinct from each other (Figure S3G). When compared to cellular levels, 932 15k EV proteins and 1517 167k EV proteins had a log2FC > 1 in comparison to cells (Figure 3H), with an overlap of 596 proteins between 15k EV and 167k EV proteins (Figure S3H). EGFR, CD9, CD63 and CD81 were all enriched in the 167k EVs compared to cellular levels (Figure 3H), which we validated by western blotting (Figure 3I). Merging of the GBM dataset to that of the DiFi dataset yielded 2229 common proteins that showed distinct abundance patterns (Figure 3J). Of the 15k EV proteins, we observed PTGFRN to be common amongst all three GBM lines and DiFi cells and DIP2A and SLC44A1 to be common amongst all four cell lines in the 167k EV category (Figure 3K).

Together these results demonstrate that GBM PDX 15k EV and 167k EV carry defined sets of proteins as cargo, that 167k EVs express canonical markers of EVs, and that few vesicles express all three tetraspanins on the same vesicle.

## 4 Proteomic Characterization of Exomeres and Supermeres Reveals Distinct Molecular Profiles in GBM

**FIGURE 4:**
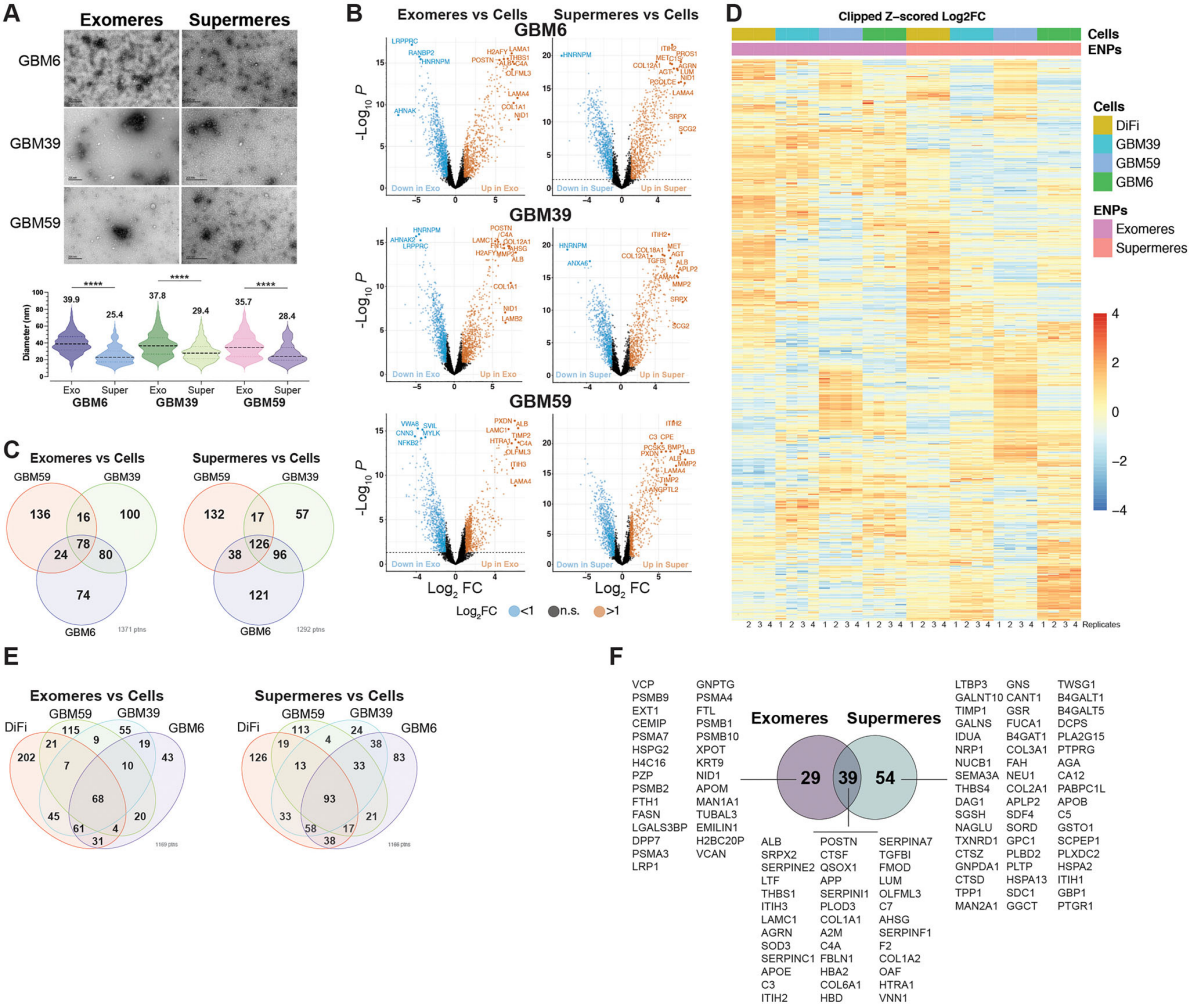
**Non‐vesicular extracellular nanoparticles have unique protein compositions. (A)** Representative TEM photomicrographs of exomeres and supermeres isolated from GBM6, GBM39 and GBM59. Below; violin plot quantitation of particle diameters using ImageJ from exomeres and supermeres of GBM6, GBM39 and GBM59 *n* = 329, 423, 336, 341, 286, 238, respectively. Data are presented as the median (black dash) and upper and lower quartiles (colored dash) of biologically independent replicates (*n* = 3), for unpaired *t* test, two‐tailed, ****p < 0.0001. **(B)** Volcano plots of proteins from GBM6, GBM39 and GBM59 exomeres and supermeres versus cells. (**C)** Venn diagram of protein species preferentially present (Log2FC > 1) in exomeres and supermeres versus cells from GBM6, GBM39 and GBM59 PDX cells. (**D)** Heat map of Log2FC > 1 of proteins in exomeres and supermeres versus cells from DiFi, GBM6, GBM39 and GBM59 cells. Data are from biologically independent replicates (*n* = 4). (**E)** Venn diagram of the overlap and uniqueness in protein species preferentially present (Log2FC > 1) in GBM and DiFi supermeres and exomeres when compared to cells. Indicated are the 10 exomere and 33 supermere proteins that are present in GBMs and absent in DiFi cells. (**F)** Venn diagram of protein species present in GBM6, GBM39, GBM59 and DiFi exomeres and supermeres.

To investigate the composition and potential roles of amembranous extracellular nanoparticles in GBM, we isolated exomeres and supermeres from the GBM PDX cell lines (GBM6, GBM39 and GBM59) using differential ultracentrifugation (Zhang et al. 2019, 2023). Exomeres were obtained by pelleting the post‐167k × *g* supernatant at 167k × *g* for 16 h, while supermeres were isolated by subjecting the exomere‐depleted supernatant to 360k × *g* for an additional 16 h (Zhang et al. 2021). TEM confirmed that supermeres were consistently smaller than exomeres across all GBM lines, with diameters ranging from 25 to 29 nm for supermeres and 35 to 40 nm for exomeres (Figure 4A). Despite their smaller size, supermeres exhibited higher protein yields per volume of conditioned media compared to exomeres and conventional EVs (15k and 167k fractions) (Figure S4A). Mass spectrometry (LC‐MS/MS) identified 6413 proteins in exomeres and 4983 in supermeres, with each nanoparticle population displaying a unique proteomic profile distinct from their respective parental cells (Figure S4B).

Proteins enriched in exomeres or supermeres (Log2FC > 1 vs. cells) were prioritized for further analysis (Figures 4B and S4C). Comparative analysis across all three GBM PDX lines revealed 78 exomere‐enriched and 126 supermere‐enriched proteins shared across the models (Figure 4C), with 47 overlapping between the two particle types (Figure S4D). To identify pan‐cancer conserved components, we also analyzed exomeres and supermeres from the CRC cell line DiFi. (Figure.S4E,F) Merging these datasets with the GBM proteomes (Figure 4D) revealed 68 and 93 proteins in exomeres and supermeres, respectively (Figure 4E), that were conserved across the two cancer types. Of these, 29 were exclusive to exomeres, while 54 were unique to supermeres (Figure 4F). GO analysis of enriched proteins showed a significant enrichment of terms related to extracellular matrix (ECM) organization and remodeling.

## 5 Distinct Functional Signatures of Exomere and Supermere Proteins

Functional annotation of particle‐enriched proteins revealed distinct profiles between exomeres and supermeres, particularly related to ECM composition and remodeling. Exomere‐specific proteins included HSPG2 (perlecan), CEMIP, EXT1, EMILIN1 and PZP—molecules known to regulate ECM structure, hyaluronan turnover, protease inhibition and TGFβ signaling (Wu et al. 2025; Muzzin et al. 2025; Busse‐Wicher et al. 2014; Yoshida et al. 2025; Cruz et al. 2020). In contrast, supermeres were enriched in a complementary but non‐overlapping set of ECM‐interacting proteins such as DAG1, APLP2, GPC1, ITIH1, B4GAT1 and LTBP3, each contributing to glycosaminoglycan biosynthesis, TGFβ regulation, or structural matrix stability (Robertson et al. 2015; Willer et al. 2014; Hamm et al. 2008; Li et al. 2020; Yanagida et al. 2023; Noell et al. 2012). Additionally, supermeres carried multiple proteases and protease regulators including TIMP1, CTSZ, CTSD and IDUA, further supporting their role in matrix remodeling (Hampe et al. 2021; Akkari et al. 2014; Vanhoutte and Heymans 2010; Ruiz‐Blazquez et al. 2024)

Several supermere‐specific proteins, such as THBS4, SDC1 and GPC1, are implicated in tumor progression, cell adhesion and intercellular signaling and have been detected in tumor‐derived EVs in previous studies. Notably, syndecan‐1 (SDC1) and glypican‐1 (GPC1) are well‐established modulators of the tumor microenvironment and are frequently associated with exosomal cargo in aggressive cancers. Importantly, some of these markers—such as GPC1 and ITIH1—were consistently enriched in supermeres derived from both GBM and CRC cells, supporting their utility as broadly conserved nanoparticle markers. However, a subset of proteins was uniquely enriched in GBM‐derived supermeres, suggesting potential CNS‐specific functions or tumor‐specific secretory programs.

Together, these findings highlight the compositional diversity of exomeres and supermeres, and establish a foundation for their functional stratification in GBM biology. Moreover, the identification of shared and tumor‐specific protein signatures offers valuable insights into their potential roles as biomarkers and therapeutic delivery platforms in brain and systemic malignancies.

### 5.1 Exomeres and Supermeres Contain Lipids

**FIGURE 5:**
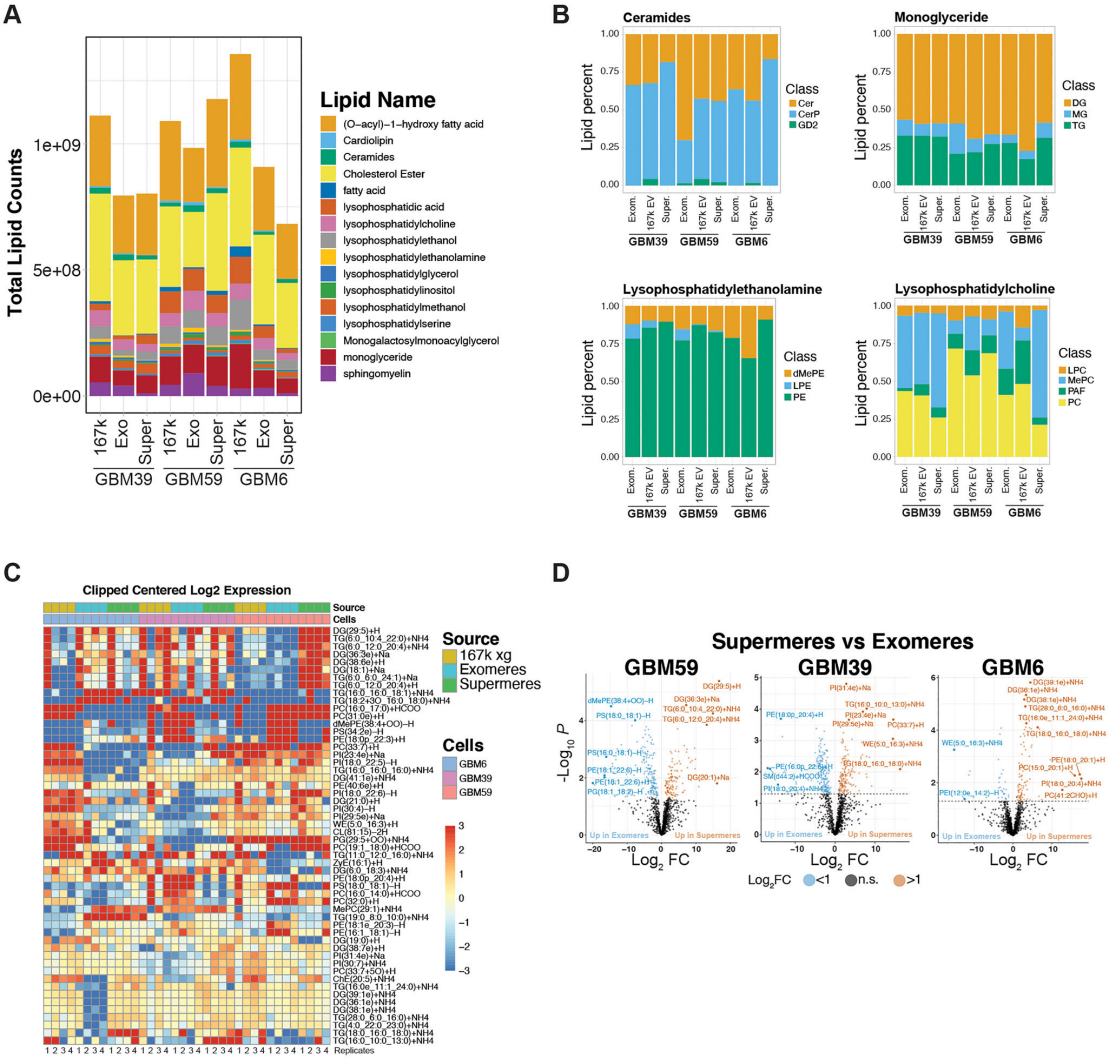
**Lipidomes of exomeres and supermeres. (A)** Total lipid counts of exomeres, supermeres and the 167k × *g* EV fractions isolated from GBM6, GBM39 and GBM39. (**B)** Relative lipid subclasses composition (percentage) for the indicated lipid classes. (**C)** Heat map of top ranked lipids of each indicated fractions. (**D)** Volcano plots of total lipids from exomeres versus supermeres in GBM6, GBM39 and GBM59.

In addition to protein, RNA and DNA, exomeres and supermeres are known to carry lipids (Zhang et al. 2018, 2021). We performed LC/MS lipidomic analysis (Breitkopf et al. 2017) of 167k EVs, exomeres and supermeres from GBM6, GBM39 and GBM59. The lipidomic profiles of exomeres and supermeres were distinct from their respective 167k EVs (Figure S5A). We detected 1610 individual lipids in all samples which categorized into 20 lipid groups and 62 subgroups (Table S2). When total lipid counts were normalized to input protein levels and compared to lipid levels in 167k EVs, total lipid counts of exomeres and supermeres in GBM6 were reduced by 1.96 ± 0.2‐fold and 2.29 ± 0.27‐fold, respectively, and by 1.49 ± 0.42‐fold and 1.56 ± 0.46‐fold in GBM39, respectively (Figure 5A). We did not observed changes in total lipid counts in the fractions of GBM59 (Figure 5A).

There are notable changes in relative composition of lipid subclasses between 167k EVs, exomeres and supermeres (Figure 5B). For example, we observed a 2.66 ± 0.17‐fold reduction in ceramide in GBM6 supermeres compared to 167k EV levels (Figure 5B), a 1.64 ± 0.31‐fold increase in GBM59 exomeres compared to 167k EV levels, and a 1.53 ± 0.04‐fold increase in ceramideP of GBM6 supermeres to 167k EV levels (Figure 5B). This indicates a shift in ceramide composition of GBM6 supermeres with a reduction in ceramide and increase in ceramideP. In monoglycerides, there was a significant 1.81 ± 0.28‐fold increase in monoglyceride in GBM6 supermeres compared to 167k EVs and a 1.82 ± 0.36‐fold increase in triglycerides in GBM6 supermeres versus 167k EVs, concomitant with a 1.31 ± 0.08‐fold decrease in monoglycerides (Figure 5B). Lysophosphatydilethanolamines were not detected in GBM6 fractions but were significantly enriched (11.85 ± 2.1‐fold) in GBM59 exomeres as compared to 167k EVs (Figure 5B). In the lysophosphatidylcholine class, we observed marked reductions of (3.72 ± 0.8 and 4.86 ± 0.3‐fold) exomeres and supermeres versus 167k EVs, respectively, in GBM6 (Figure 5B). In GBM6 supermeres, there was a significant 8.27 ± 0.21‐fold increase in MePC versus 167k EV and a commensurate 5.98 ± 0.45‐fold reduction in PAF and 2.28 ± 0.2‐fold reduction in PC in comparison to 167k EVs (Figure 5B).

On a single lipid basis, when compared to each other, exomeres and supermeres have noteworthy differences, with several species of DG being highly represented in supermeres (Figure 5C,D). Taken together, these results demonstrate specific changes in lipid compositions between particles and in between the GBM PDX lines.

### 5.2 EVs and NVEPs Transport Distinct sRNAs

**FIGURE 6:**
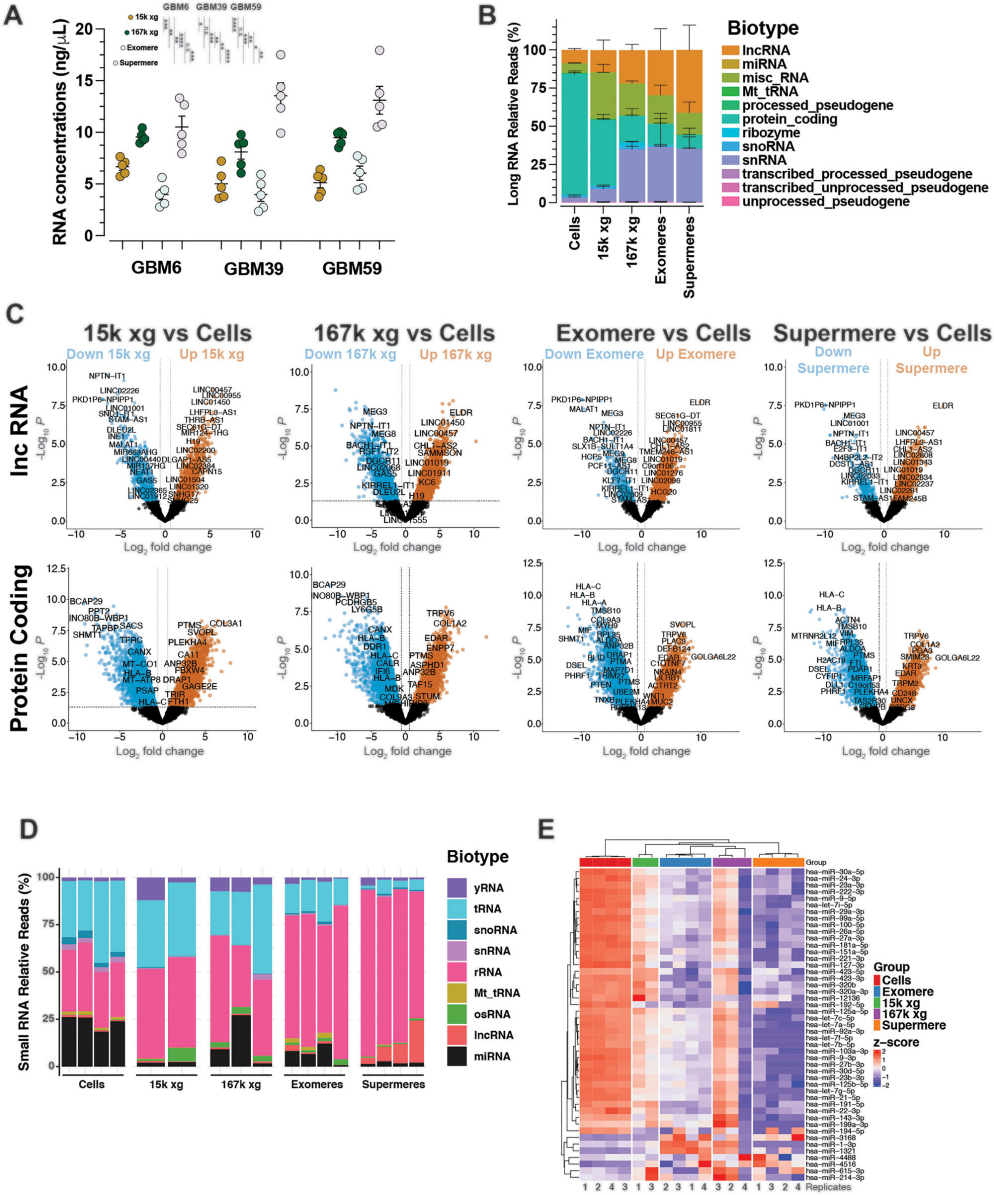
**RNA distribution and composition of EVs and NVEPs. (A)** Relative abundance of RNA isolated from 15k EVs, 167k EVs, exomeres and supermeres from GBM6, GBM39 and GBM59 PDX cells. Data are presented as the mean ± SEM of biologically independent replicates (*n* = 5), unpaired *t* test, two‐tailed, *p < 0.05, **p < 0.01, ***p < 0.001 and ****p < 0.0001. (**B)** Relative long‐RNA read counts (percentage) of the indicated RNA biotypes from RNAseq of cells, 15k EVs, 167k EVs, exomeres and supermeres of GBM39. Note that Misc_RNAs are mainly composed of RNA 7SL cytoplasmic pseudogenes, RN7SK pseudogenes, vault RNAs and Y RNAs. Data are presented as the mean ± SEM of biologically independent replicates (*n* = 4). (**C)** Volcano plots of log2FC abundance differences between the indicated EVs and NVEPs versus cells from GBM39 for the indicated long RNA biotypes. (**D)** Relative small‐RNA read counts of the indicated RNA biotypes from RNAseq of cells, 15k EVs, 167k EVs, exomeres and supermeres of GBM39. Data are presented as the mean ± SEM of biologically independent replicates (*n* = 2–4). yRNA, Y RNA‐derived sRNA; tRNA, tRNA‐derived sRNA; snoRNA, small nucleolar RNA‐derived sRNA; snRNA, small nuclear RNA‐derived sRNA; rRNA ribosomal RNA‐derived sRNA; Mt_tRNA, mitochondrial tRNA‐derived sRNA; osRNA, other sRNA; lncRNA, long non‐coding RNA‐derived sRNA; and miRNA, microRNA. (**E)** Heatmap of the top‐50 most abundant miRNAs across GBM39 cells and the indicated extracellular compartments.

Vesicular and non‐vesicular extracellular RNA (exRNA) molecules have gained significant interest because of their diverse biological roles and potential as cancer biomarkers (Jeppesen et al. 2019; Murillo et al. 2019). We analyzed the RNA content of cells and extracellular carriers. We isolated total RNA from cells and from their respective UC‐isolated 15k EV, 167k EV, exomere and supermere fractions. We observed higher amounts of RNA isolated from supermeres than exomeres, 15k EVs, or 167k EVs (Figure 6A). However, upon normalization to protein content, no differences in extracellular RNA levels were observed between any of the compartments (Figure S6A).

Next, we isolated long and small non‐coding RNAs (sRNA) from cells, EVs and NVEPs derived from GBM39, and performed bulk RNAseq. The long exRNAs associated with GBM39 cells and their extracellular compartments displayed distinct patterns of distribution (Figures 6B and S6B,C) and unique species are differentially abundant amongst cells, EVs and NVEPs (Figure S6D). The major RNA biotypes detected in cells are protein‐coding RNAs and long non‐coding RNA (lncRNAs) making up 79.28% and 8.41% of reads, respectively (Figures 6B and S6C, Table S3). The major RNA biotypes found in 15k EVs are protein‐coding RNAs, miscellaneous RNAs and lncRNAs making up 42.82%, 30.46% and 14.52% of reads, respectively (Figures 6B and S6C, Table S3). The composition of RNA biotypes in 167k EVs, on the other hand, drastically differ from cells and 15k EVs, with full‐length small nuclear RNA (snRNA), lncRNA, miscellaneous RNA and protein‐coding RNAs making up 34.11%, 21.72%, 20.94% and 17.15%, respectively. Exomeres and supermeres are mostly composed of full‐length snRNAs (35.72% and 34.85%, respectively), lncRNA (29.54% and 41.11%, respectively), miscellaneous RNA (18.45% and 14.34%, respectively) and protein‐coding RNAs (13.53% and 8.47%, respectively) (Figures 6B and S6C, Table S3). Mitochondrial tRNA, pseudogenes, ribozyme and full‐length snoRNAs were detected at low levels, partly due to the enhancement for longer RNA during the isolation procedures. When compared to cells, the relative levels of protein‐coding exRNAs are significantly reduced in 15k EVs by 1.9‐fold ± 0.09 (Figures 5B and S5C, and Table S3), and 167k EVs contain relatively less protein‐coding RNAs than cells (4.6‐fold reduction ± 0.29) and 15k EVs (2.5‐fold reduction ± 0.3). Exomeres and supermeres contain lower levels of protein‐coding RNAs with fold reductions of 5.9 ± 0.54 and 9.4 ± 0.55 when compared to cells, respectively. On the other hand, both 15k and 167k EVs display significant increased levels of miscellaneous RNAs when compared to cells (4.8 ± 0.17 and 3.3 ± 0.08, respectively) (Figures 6B and S6C, Table S3). Similarly, significant increases in snRNA in 15k EVs, 167k EVs, exomeres and supermeres were observed when compared to cellular levels (fold‐increases 3.0 ± 0.27, 12. 4 ± 0.2, 12.9 ± 0.54 and 12.6 ± 0.27, respectively), and a trend for higher read counts of lncRNA in 15k EVs, 167k EVs, exomeres and supermeres was found; however, most did not reach statistical significance due to outliers in the replicates (Figures 6B and S6C, Table S3).

Analyzing abundance differences in lncRNAs and protein‐coding RNAs between 15k EV, 167k EV, exomeres and supermeres when compared to cells revealed lncRNA and mRNA species that are preferentially overrepresented and underrepresented in EVs and NVEPs (Figure 6C, Table S4). Although a significant overlap exists amongst these species, there are lncRNAs and mRNAs that are unique to 15k EVs, 167k EVs, exomeres and supermeres (Figure S6E).

For sRNAs, cells were found to be enriched with miRNAs, and sRNAs‐derived from parent tRNAs and rRNAs (Figure 6D). The majority class of sRNAs on EVs and NVEPs were rRNA‐derived sRNAs. Some miRNAs were found associated with 15k EVs and exomeres; however, supermeres and 15k EVs were not found to detect large percentages of miRNAs compared to other sRNAs classes (Figure 6D). Nevertheless, supermere miRNA content was found to be most distinct from that of parent cells (Figure 6E).

### 5.3 Supermeres Cross the BBB

**FIGURE 7:**
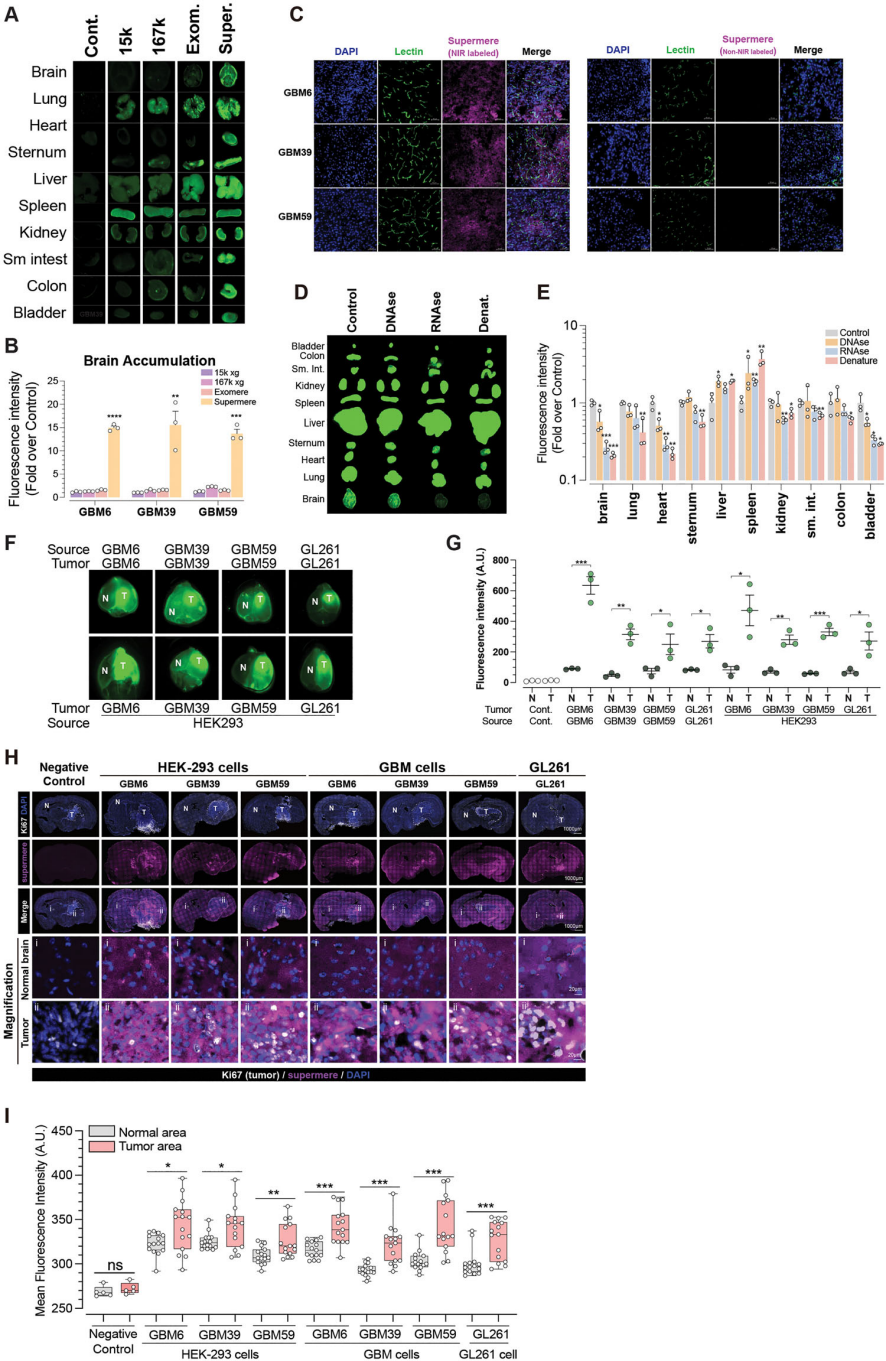
**Supermeres exhibit preferential CNS tissue uptake in vivo. (A)** Representative NIR images of whole‐organ imaging. Male CD‐1 mice (*n* = 3) were injected (IV) with 150 µg (protein) (in 200 µL of PBS) of NIR‐labelled 15k EVs, 167k EVs, exomeres or supermeres derived from GBM6, GBM39 and GBM59 cells and the indicated organs were harvested after 24 h and imaged. (**B)** Brain accumulation of EVs and NVEPs. Quantitation of brain signal intensity of the indicated EVs and NVEPs labeled with NIR, 24 h post IV tail vein injected. Data are presented as the mean ± SEM of biologically independent replicates (*n* = 3), unpaired t test, two‐tailed, **p < 0.01, ***p < 0.001 and ****p < 0.0001. (**C)** Extravasation of supermeres into brain parenchyma. 150 µg (protein) (in 200 µL of PBS) NIR‐labeled GBM6, GBM39 and GBM59 supermeres were IV (tail vein) injected and 6 h later, fluorescently labeled lectin (5 mg/kg) was administered via tail vein injection 30 min prior to mouse euthanasia. (**D, E)** Whole‐organ imaging. Representative NIR images of organs (D) and quantitation (E) of NIR‐labeled GBM39 supermeres pretreated with DNase, RNase, or heat denatured IV injected in CD1 male mice and organ‐harvested and imaged 24 h post‐injections. 150 µg (protein) (in 200 µL of PBS) were injected. Data are presented as the mean ± SEM of biologically independent replicates (*n* = 3), unpaired *t* test, two‐tailed, *p < 0.05, **p < 0.01, and ***p < 0.001. (**F, G)** Representative photomicrographs of NIR image of GBM6, GBM39, GBM59 and GBL261 tumor‐bearing mouse brain. Mouse brains were harvested after 24 h of 150 ug (protein) (in 200 µL of PBS) of GBM6, GBM39 and GBM59 and HEK‐293 and GL261 NIR‐labeled supermeres IV injection (F) and quantitation (G). Data are presented as the mean ± SEM of biologically independent replicates (*n* = 3), unpaired *t* test, two‐tailed, *p < 0.05, **p < 0.01 and ***p < 0.001. (**H, I)** Representative photomicrographs (H) and quantification of signal intensity (I) of tumor‐bearing‐mouse brain tissue sections that were processed for immunofluorescence staining against Ki‐67 to identify GBM cells and imaged to detect NIR‐labeled supermeres. Scale bars = 1000 µm and 20 µm. Data are presented as the mean ± SEM of biologically independent replicates (*n* = 3), unpaired *t* test, two‐tailed, **p < 0.01, **p < 0.01, and ***p < 0.001.

We next investigated the in vivo uptake and biodistribution of EVs and NVEPs. Initial experiments established that intravenous (IV) delivery of near‐infrared (NIR) dye–labeled supermeres via the tail vein resulted in more efficient and widespread organ distribution compared to intraperitoneal administration (Figure S7A). We then covalently labeled 15k EVs, 167k EVs, exomeres and supermeres (normalized to total protein) derived from GBM6, GBM39 and GBM59 cells with NIR dye and injected them IV into C57Bl6/J mice (*n* = 3). Organs were harvested 24 h post‐injection, imaged and signal intensity quantified (Figures 7A,B and S7B). Among the particle types, 15k EVs accumulated primarily in the lungs, liver, spleen and kidneys, whereas 167k EVs exhibited a broader biodistribution, with additional accumulation in the heart, small intestine, colon and bladder (Figures 7A,B and S7B). Exomeres displayed the most restricted distribution, localizing mainly to lung and liver. In contrast, supermeres demonstrated the widest and most intense organ accumulation profile (Figures 7A,B and S7B), including robust and statistically significant uptake in brain tissue. Specifically, supermeres from GBM6, GBM39 and GBM59 displayed 15.0 ± 0.06, 15.5 ± 0.33 and 13.7 ± 0.13‐fold enrichment in brain relative to controls, respectively (Figure 7B). To assess whether NIR‐labeled supermeres cross the blood–brain barrier and extravasate to the brain parenchyma, we labeled brain vasculature with a fluorescent lectin following IV injection of supermeres and performed confocal microscopy. This analysis revealed supermere localization outside the vasculature and within the parenchyma (Figure 7C).

To identify which of these biomolecular components are required for the supermeres’ ability to penetrate the CNS, we treated GBM39‐derived supermeres with DNase, RNase, or subjected them to protein denaturation (Figure S7C) and subsequently assessed their biodistribution in vivo (Figure 7D,E). DNase treatment had minimal impact on supermere uptake across organs, including the brain (Figure 7D,E). In contrast, RNase treatment or heat‐induced protein denaturation markedly and significantly impaired CNS accumulation, reducing brain uptake by 3.8 ± 0.2‐fold and 4.8 ± 0.1‐fold, respectively (Figure 7D,E). Similar decreases were also observed in the heart (Figure 7D,E), suggesting that both RNA integrity and protein conformation are critical for supermere biodistribution.

We next exploited the intrinsic BBB‐penetrating ability of supermeres to determine whether they can localize to brain tumors. GBM‐bearing mice were generated by stereotactic intracranial implantation of GBM6, GBM39 and GBM59 cells into immunocompromised Ncr^Nu/Nu^ mice. Fourteen days post‐implantation, animals were injected intravenously with NIR‐labeled supermeres derived from GBM6, GBM39, GBM59 or HEK‐293 cells and analyzed 24 h later for organ (Figure S7D,E) and brain distribution (Figure 7F,G). To assess whether host immune status influenced tumor targeting, the same experiments were performed in the GL261 syngeneic GBM model. In both models, supermeres accumulated at significantly higher levels within GBM tumor tissue compared to adjacent non‐tumor CNS regions (Figure 7F,G). Confocal microscopy confirmed preferential localization of supermeres within tumor tissue relative to surrounding normal brain parenchyma (Figure 7H,I).

Collectively, these findings show that IV‐administered supermeres efficiently cross the BBB, accumulate within normal CNS tissue and preferentially localize to brain tumor sites, with this targeting critically dependent on the preservation of both their RNA and protein integrity.

### 5.4 Supermeres Repress TGFB Anti‐Inflammatory Pathways in Microglia

**FIGURE 8:**
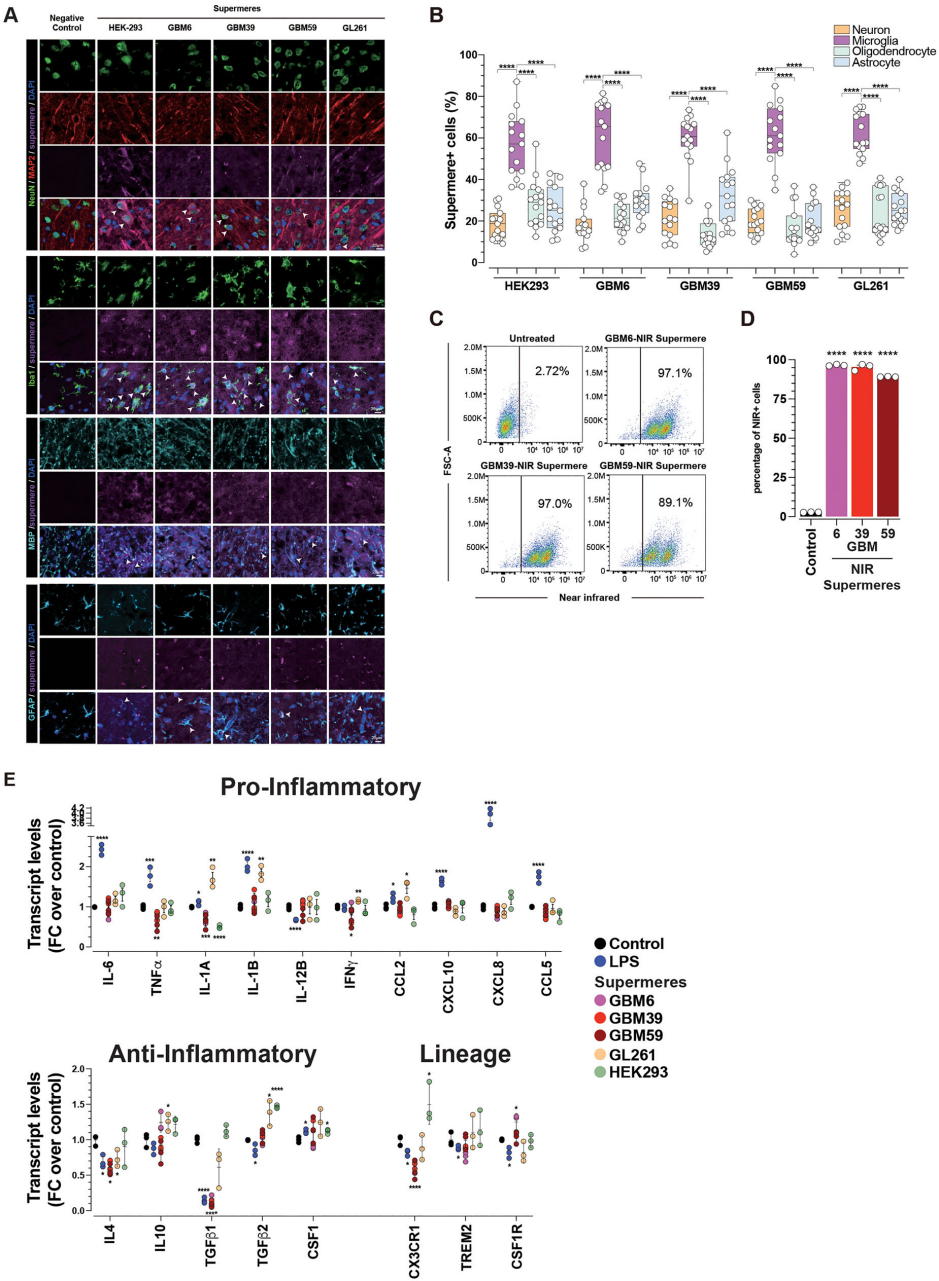
**Supermeres activate microglia. (A)** Representative photomicrographs of immunofluorescence of mouse brain sections of mice IV injected with 150 µg (protein) (in 200 µL of PBS) of NIR‐labeled supermeres isolated from the indicated cells and imaged 48 h later, processed for brain sections and immunofluorescence to identify neuron (MAP2 and NeuN), microglia (IBA1), oligodendrocytes (MBP) and astrocytes (GFAP). Scale bars = 20 µm. (**B)** Quantitation (percentage) of indicated cell types positive for supermeres. Data are presented as the mean ± SEM of biologically independent replicates (*n* = 3), unpaired *t* test, two‐tailed, ****p < 0.0001. (**C, D)** Representative flow cytometry plot (C) and quantitation (D) of HMC3 microglial cells treated with 50 µg/mL of GBM6, GBM39, and GBM59 PDX cells NIR‐labeled supermeres for 6 h and analyzed. Data are presented as the mean ± SEM of biologically independent replicates (*n* = 3), unpaired *t* test, two‐tailed, ****p < 0.0001. (**E)** qRT PCR of the indicated genes of RNA isolated from HMC3 cells treated with LPS (100 ng/mL), and 50 µg/mL of GBM6, GBM39, GBM59 PDX, GL261 and HEK‐293 cells supermeres for 6 h. Data are presented as the mean ± SEM of biologically independent replicates (*n* = 3), unpaired *t* test, two‐tailed, *p < 0.05, **p < 0.01, ***p < 0.001 and ****p < 0.0001.

Next, we determined the types of cells that uptake supermeres in the CNS. NIR‐labeled supermeres isolated from HEK‐293, GBM6, GBM39, GBM59 and GL261 were tail‐vein injected in mice and 48 h later, mice were sacrificed and processed for colocalization with cell type‐specific markers using indirect immunofluorescence microscopy. Uptake of supermeres by microglia in vivo was significantly higher than in neurons, astrocytes and oligodendrocyte cells (Figure 8A,B).

Microglia can rapidly adapt to changes in their microenvironment by expressing genes associated with either pro‐ or anti‐inflammatory responses (Paolicelli et al. 2022). To determine the effects of supermeres on microglia, we studied microglia polarization in vitro. We first determined that the human microglia cell line, HMC3 (Cappoli et al. 2018), can uptake supermere efficiently (Figure 8C,D), and the responses to supermeres were concentration‐ and time‐dependent (Figure S8A,B). HMC3 cells incubated with supermeres displayed changes in expression of genes involved in microglial polarization. We performed qRT‐PCR on a gene panel to determine the biological consequences of supermeres on microglia. HMC3 microglia cells were treated with LPS‐free supermeres (Figure S8C) isolated from GBM6, GBM39, GBM59, HEK‐293 and GL261 cells. Bacterial lipopolysaccharide (LPS) treatment was used as a positive control for pro‐inflammatory responses. Incubation of HMC3 cells with GBM6, GBM39 and GBM59 supermeres induced significant reduction in the mRNA levels of anti‐inflammatory polarization markers TGFβ1 and IL‐4 to a similar extent as LPS stimulation (Figure 8E). This effect was not paralleled with HEK‐293 supermere incubation (Fig 8E). Interestingly, HEK‐293 supermeres triggered an increase in TGFβ2 mRNA levels (Figure 8E). We also observed significant but small decreases in expression of TNFa and IL‐1A upon GBM6, GBM39 and GBM59 supermeres stimulation (Figure 8E). The decrease in TNFa mRNA levels was not observed with GL261 or HEK‐293 supermere incubation (Figure 8E). However, treatment with HEK‐293 supermeres reduced IL‐1A mRNA levels to a similar extent as GBM6, GBM39 and GBM59 supermeres whereas incubation with GL261 supermeres triggered an increase (Figure 8E). Together, these results demonstrate that supermeres preferentially interacts with microglia and this interaction triggers a reduction in TGFβ1 expression, a known anti‐inflammatory response gene.

## 6 Discussion

The extensive heterogeneity in size and molecular composition of EVs and non‐vesicular extracellular particles (NVEPs) remains a fundamental challenge in the field of extracellular communication (Carney et al. 2024). In this study, we applied multi‐omics approaches to characterize four classes of extracellular particles that can be efficiently isolated using simple and cost‐effective differential centrifugation. Consistent with Stokes’ law, particle sedimentation under centrifugal force depends primarily on size and density. Since 15k and 167k EVs are similar in size (Figure 1), we hypothesized that differences in density account for their distinct sedimentation profiles. Our findings support this notion: 15k EVs are significantly denser than 167k EVs, as evidenced by their higher protein content—approximately eight‐fold greater per vesicle—as well as their distinct and more lipid‐rich composition. These data suggest that the higher density of 15k EVs facilitates their recovery at lower centrifugal speeds relative to 167k EVs.

Proteomic profiling further reinforced the biological distinction between these vesicle populations. Global proteomics and confirmatory western blotting revealed that 167k EVs are enriched with classical small EV markers, including the tetraspanins CD9, CD63 and CD81 (Figure 3I), consistent with their derivation from the endosomal system and multivesicular body (MVB) pathway (Thery et al. 2002). In contrast, 15k EVs likely originate from plasma membrane shedding (Cocucci and Meldolesi 2015) or alternative, non‐canonical, ESCRT‐independent endosomal mechanisms (Colombo et al. 2014). These ontological differences were further supported by the identification of distinct protein signatures for each EV subtype, enabling more refined classification and providing mechanistic insight into their biogenesis.

Among the proteins consistently enriched in 15k EVs across all three GBM PDX lines, Myeloid‐Associated Differentiation Marker (MYADM) emerged as a particularly informative marker. MYADM is a membrane‐associated protein implicated in cytoskeletal organization, membrane domain regulation and lipid raft formation (Labat‐de‐Hoz et al. 2023, Aranda et al. 2011). Lipid rafts, cholesterol‐ and sphingolipid‐rich domains of the plasma membrane, are well‐established contributors to vesicle formation and release, particularly for non‐endosomally derived EVs. The presence of MYADM in 15k EVs thus supports a plasma membrane shedding origin and may serve as a functional marker for this class of EVs in GBM. These findings collectively deepen our understanding of EV heterogeneity and underscore the importance of integrating physical properties with molecular content to delineate distinct extracellular particle populations.

To refine our identification of subtype‐specific markers for EVs, we integrated proteomic datasets from both GBM PDX models and the CRC cell line DiFi. This comparative analysis led to the identification of Prostaglandin F2 Receptor Negative Regulator (PTGFRN, also known as CD315) as a candidate marker enriched in the 15k EV fraction. PTGFRN is a member of the immunoglobulin superfamily and is known to associate with tetraspanins CD9 and CD81 (Hemler 2005; Charrin et al. 2009, 2014) via their transmembrane domains (Oosterheert et al. 2020; Charrin et al. 2009; Andre et al. 2009), contributing to the architecture of tetraspanin‐enriched microdomains (TEMs), which play critical roles in membrane organization, cell adhesion and migration. Recent studies have highlighted PTGFRN as an EV‐associated protein of particular interest. In HeLa cells, PTGFRN has been identified as a marker of vesicles budding directly from the plasma membrane (Mathieu et al. 2021), rather than from canonical endosomal pathways. Moreover, PTGFRN has been shown to serve as a versatile scaffold for engineering surface display and luminal loading of cargo in EVs (Dooley et al. 2021), broadening its potential utility in both diagnostic and therapeutic applications. Our findings build on this emerging evidence and suggest that PTGFRN may serve as a pan‐cancer marker of EVs derived through non‐canonical, ESCRT‐independent pathways—distinct from the classical CD9‐, CD63‐, or CD81‐marked small EVs.

In contrast, proteins enriched in the 167k EV fraction across both GBM and DiFi cell lines included Disco‐Interacting Protein 2 Homolog A (DIP2A), a transmembrane protein with established roles in neuronal development and synaptic maintenance (Zhang et al. 2023). Although DIP2A has not yet been functionally linked to EV biology, its consistent presence in the 167k EV proteome warrants further investigation into its potential roles in vesicle biogenesis or cargo specificity. Additionally, we identified Solute Carrier Family 44 Member 1 (SLC44A1, also known as CTL1), a choline transporter implicated in membrane phospholipid synthesis. SLC44A1 has recently been detected in EV proteomes (Fan et al. 2023) and may contribute to vesicle formation through its role in lipid metabolism. Together, these findings expand the repertoire of EV‐associated proteins and reinforce the molecular diversity and mechanistic complexity underlying EV biogenesis across different tumor types.

Our proteomic profiling of exomeres and supermeres in GBM provides new insight into the molecular heterogeneity of amembranous extracellular nanoparticles and their potential functional specializations. The observation that supermeres are smaller yet more protein‐rich than exomeres, while harboring distinct and largely non‐overlapping protein repertoires, underscores that these particle types likely arise from different biogenetic pathways and serve complementary biological roles. The strong enrichment of extracellular matrix–associated proteins in both populations’ points to a shared role in ECM remodeling, a process central to GBM invasion and progression. However, the unique enrichment of glypican‐1, syndecan‐1, ITIH1 and other tumor‐promoting factors in supermeres, many of which were conserved between GBM and CRC, suggests that supermeres may serve as broadly conserved vehicles for modulating the tumor microenvironment across cancer types. At the same time, the detection of GBM‐specific supermere cargo highlights the possibility of CNS‐ or tumor‐specific signaling functions that could be leveraged for biomarker discovery or targeted therapy. Collectively, these findings not only expand our understanding of the molecular complexity of extracellular nanoparticles in GBM but also identify candidate molecules and pathways that may be exploited for diagnostic and therapeutic applications.

Our RNA sequencing analysis of both long and small RNA species revealed striking differences in RNA subtype distribution among EVs and NVEPs. Notably, protein‐coding RNAs were enriched in 15k EVs relative to 167k EVs, exomeres and supermeres. In contrast, lncRNAs and snRNAs were found at lower abundance in 15k EVs but were more prevalent in 167k EVs, exomeres and supermeres. Interestingly, the RNA subtype profiles of exomeres and supermeres were largely similar, suggesting potential functional or biogenetic parallels between these two NVEP populations. Prior studies have shown that the longest intact mRNA sequences identified in EV preparations are typically under ∼1 kilobase in length, indicating extensive fragmentation of RNA cargo within extracellular particles (Wei et al. 2017; Hinger et al. 2020, 2018). These findings support the idea that the majority of RNA molecules found in EVs and NVEPs are unlikely to be functional in their full‐length form, thereby limiting their direct coding potential. This raises the possibility that the inclusion of fragmented RNA in EVs and NVEPs may reflect a selective cellular mechanism for the disposal of surplus or degraded RNA transcripts, rather than a mechanism for functional RNA delivery. Whether recipient cells possess the capability to process and utilize these RNA fragments functionally upon uptake remains an open and compelling question. Future studies examining the fate of these RNAs post‐transfer and their potential roles in modulating recipient cell biology will be crucial to fully elucidate the biological significance of RNA cargo in extracellular communication.

Our biodistribution studies using NIR‐labeled extracellular particles revealed significant differences in organ uptake profiles between EVs and NVEPs. Following systemic administration via tail‐vein injection, 15k EVs, 167k EVs and exomeres demonstrated minimal biodistribution, with limited accumulation observed primarily in the liver and lungs (Figure 7). In stark contrast, supermeres exhibited robust uptake across multiple organs, with particularly striking accumulation in the brain—showing a 15‐fold increase compared to 15k EVs, 167k EVs and exomeres, underscoring the unique ability of supermeres to traverse the BBB. These observations are consistent with earlier reports demonstrating the CNS‐targeting potential of supermeres (Zhang et al. 2018, 2021). Our findings suggest that the efficient BBB penetration of supermeres is not solely attributable to their small size. Rather, their structural integrity, including both conformational features and intact RNA‐protein composition, appears to be essential for CNS entry. Disruption of either component compromises this ability, indicating that the cargo and molecular architecture of supermeres contribute directly to their biodistribution properties.

Notably, supermeres also accumulate in GBM tumors following systemic injection, highlighting their potential utility as delivery vehicles for CNS‐targeted therapies. This tumor‐targeting phenomenon was also observed with supermeres derived from non‐cancerous HEK‐293 cells, and occurred in both immunocompetent and immunodeficient mouse models. These data suggest that supermere homing to GBM is independent of host immune status and may be driven by conserved features of the tumor microenvironment and/or the supermeres themselves. Within the CNS, supermeres exhibit a pronounced tropism for microglia, the resident innate immune cells. The precise mechanisms by which supermeres interact with microglia remain to be elucidated. One plausible explanation is active uptake via phagocytosis, given the high phagocytic capacity of microglia. Alternatively, supermeres may serve as damage‐associated molecular patterns (DAMPs), activating pattern recognition receptors (PRRs) such as Toll‐like receptors (TLRs). In this context, we found that treatment of microglial cells with GBM‐derived supermeres led to a marked reduction in anti‐inflammatory cytokine production—most notably IL‐4 and TGFβ1—mimicking the effects observed with lipopolysaccharide (LPS), a prototypical TLR4 ligand. This suggests that GBM‐derived supermeres interact with Toll‐like receptors (TLRs), likely TLR4, as LPS‐mediated reductions in TGFβ transcript levels are known to occur through TLR4 engagement (Suga et al. 2014).

Interestingly, while both GBM‐ and HEK‐293‐derived supermeres interacted with microglia, only the former significantly reduced TGFβ1 transcript levels. This suggests that tumor‐derived supermeres possess unique bioactive properties, possibly conferred by distinct surface receptor or cargo profiles. It is also conceivable that differential intracellular trafficking or signaling occurs following uptake, resulting in distinct immune‐modulatory outcomes. Together, these findings support a model in which supermeres not only efficiently target the brain and GBM tissue but also modulate microglial function, potentially through both receptor‐mediated signaling and cargo delivery. Further mechanistic studies are warranted to dissect the specific components and pathways responsible for these effects, which could ultimately enable the rational design of supermere‐based nanotherapeutics for brain cancer and other CNS pathologies.

In conclusion, the distinct RNA and protein compositions of EVs reflect their endosomal or plasma membrane origins, while the biogenesis of amembranous NVEPs such as supermeres remains to be fully defined. Among these, supermeres exhibit a unique capacity to cross the BBB following systemic administration and engage microglial cells to elicit immunomodulatory effects. Specifically, supermeres attenuate anti‐inflammatory signaling in microglia, potentially shifting the CNS immune milieu toward a less immunosuppressive, tumor‐permissive state. To enhance their utility as therapeutic delivery vectors for brain tumors and other CNS diseases, future studies should explore alternative delivery routes, such as intra‐carotid administration, to improve targeting while limiting systemic exposure. These findings underscore the promise of supermeres as a novel platform for CNS‐targeted therapies and warrant further mechanistic and translational investigation.

### 6.1 Resource Availability

#### 6.1.1 Lead Contact

Further information and requests for resources and reagents should be directed to and will be fulfilled by the lead contact, Al Charest (acharest@bidmc.harvard.edu).

#### 6.1.2 Materials Availability

Presented materials are made available upon reasonable request to the lead contact.

#### 6.1.3 Data and Code Availability

Data: Proteomics data are available at ProteomeXchange.org under project name “Diversity and Heterogeneity of EVs and Brain‐Penetrating Amembranous Non‐Vesicular Nanoparticles in Glioblastoma”, with identifier: PXD059671. RNAseq data are available at GEO, accession numbers: GSE272370, and GSE286513

Code: No code was generated in this study. Any additional information required to reanalyze the data reported in this paper is available from the lead contact upon request.

### 6.2 Limitations of the Study

Although our findings underscore the unique potential of supermeres in CNS‐targeted delivery, several limitations should be acknowledged. First, the precise mechanisms by which supermeres traverse the BBB remain undefined. Although our data suggest that both structural integrity and RNA content are critical for CNS penetration, we were unable to fully disentangle the relative contributions of these factors. For instance, heat denaturation disrupts both protein conformation and RNA integrity, whereas RNase treatment selectively degrades RNA; both interventions significantly impaired BBB crossing. However, these approaches do not definitively distinguish whether loss of function is due to disruption of secondary structure, RNA‐specific signaling, or the destruction of essential scaffolding components. Additionally, the biogenesis and molecular determinants governing the assembly of exomeres and supermeres remain incompletely characterized. A deeper understanding of their structural organization and molecular constituents will be necessary, particularly if supermeres are to be optimized as vehicles for therapeutic delivery to the CNS. Future studies employing targeted mutagenesis, structural biology and biophysical approaches will be essential to address these questions.

## Author Contributions

**Tuoye Xu**: methodology, formal analysis, investigation. **Joao A. Paulo**: methodology, formal analysis, investigation. **Piyan Zhang**: methodology, formal analysis, investigation. **Xinyue Liu**: methodology, formal analysis, investigation. **Alya Nguyen**: methodology, formal analysis, investigation. **Yuanhua Cheng**: writing – review and editing. **Clark Massick**: methodology, investigation, formal analysis. **Yanhong Zhang**: methodology, investigation. **Dennis K. Jeppesen**: writing – review and editing. **Qin Zhang**: writing – review and editing. **James N. Higginbotham**: investigation. **Oleg S. Tutanov**: investigation. **Anna M. Krichevsky**: investigation. **Daniel T. Chiu**: writing – review and editing, supervision. **Steve P. Gygi**: supervision. **Kasey C. Vickers**: conceptualization, formal analysis, writing – review and editing, supervision. **Jeffrey L. Franklin**: conceptualization, writing – review and editing, supervision. **Robert J. Coffey**: conceptualization, writing – review and editing, funding acquisition, supervision. **Al Charest**: conceptualization, methodology, formal analysis, writing – original draft, writing – review and editing, funding acquisition, supervision.

## Conflicts of Interest

The authors declare no competing interests.

## Supporting information



Supplementary material

Supplementary material

## Data Availability

Proteomics data files are available within the supplementary data. RNAseq and sRNA‐seq fastq files and processed data are available through GEO.
