# Supplementary material for: Heterogeneity of Extracellular Vesicles and Non‐Vesicular Nanoparticles in Glioblastoma
Source: J Extracell Vesicles. 2025 Oct 2;14(10):e70168. doi: 10.1002/jev2.70168 (PMC12491655; doi:10.1002/jev2.70168)
Supplement: Supplementary file 1 — Supplementary material [file JEV2-14-e70168-s002.docx]

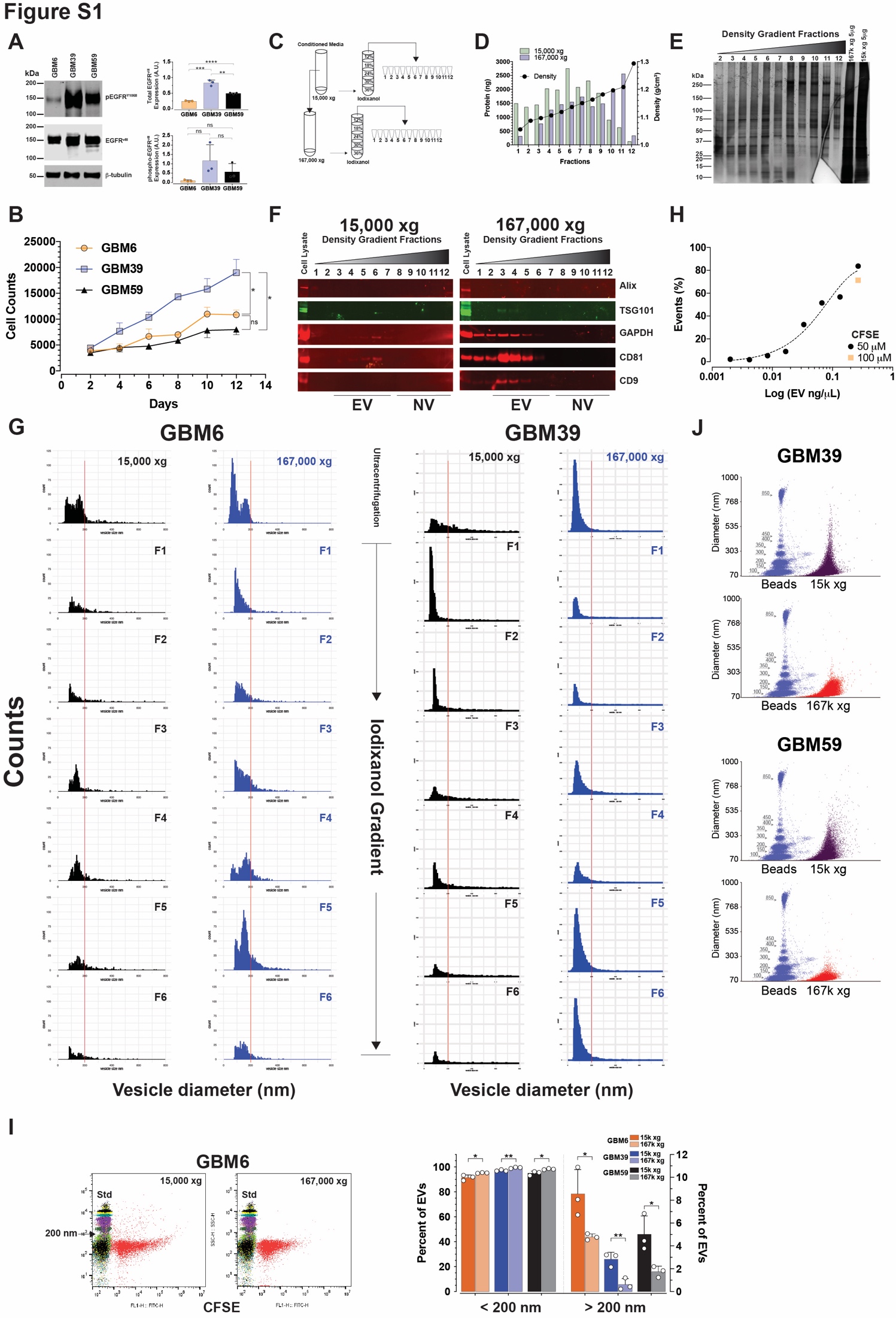


**Fig. S1. Size characterization of EVs isolated by differential UC. A)** Representative quantitative western blot of cellular activated phospho-EGFR and total EGFR from the indicated GBM PDX cell cultures. Data are presented as the mean ± S.D. of biologically independent replicates, n=3, unpaired t test, two-tailed, **p<0.01, ***p<0.001 and ****p<0.0001. **B)** Growth rates of the indicated GBM PDX cell cultures. Data are presented as the mean ± S.E.M. of biologically independent replicates, unpaired t test, two-tailed, *p<0.05. **C-F)** Graphical representation of the iodixanol density gradient ultracentrifugation (DGUC) (C), yield in total protein of the fractions from the DGUC for 15k x*g* and 167k x*g* fractions along with their densities (D), silver stained SDS-PAGE of the DGUC fractions (E), and western blotting of each fractions from 15k x*g* and 167k x*g* fractions for the indicated EV markers (F) for GBM-6. **G)** Quantification of TEM images of 15k x*g* and 167k x*g* EVs from DGUC fractions 1-6 for GBM-6 and GBM-39. **H,I)** Dose response of carboxyfluorescein succinimidyl ester (CFSE), which covalently labels molecules with the fluorescent carboxyfluorescein dye, binding to increasing concentrations of EVs as determined by total protein content (ng/mL) (H), representative flow cytometry of 15k x*g* and 167k x*g* EVs size determination using CFSE dye staining (left) and quantitation of percentage of 15k EVs and 167k EVs above or below a threshold of 200 nm diameter for the indicated GBM PDX cell cultures. Data are presented as the mean ± S.D. of biologically independent replicates, n=3, unpaired t test, two-tailed, *p<0.05, **p<0.01 (I). **J)** Representative flow cytometry plot of vesicle size from differential UC 15k x*g* and 167k x*g* isolated EVs from GBM-39 and -59 PDX cell culture. Bead marker sizes are indicated in nm.


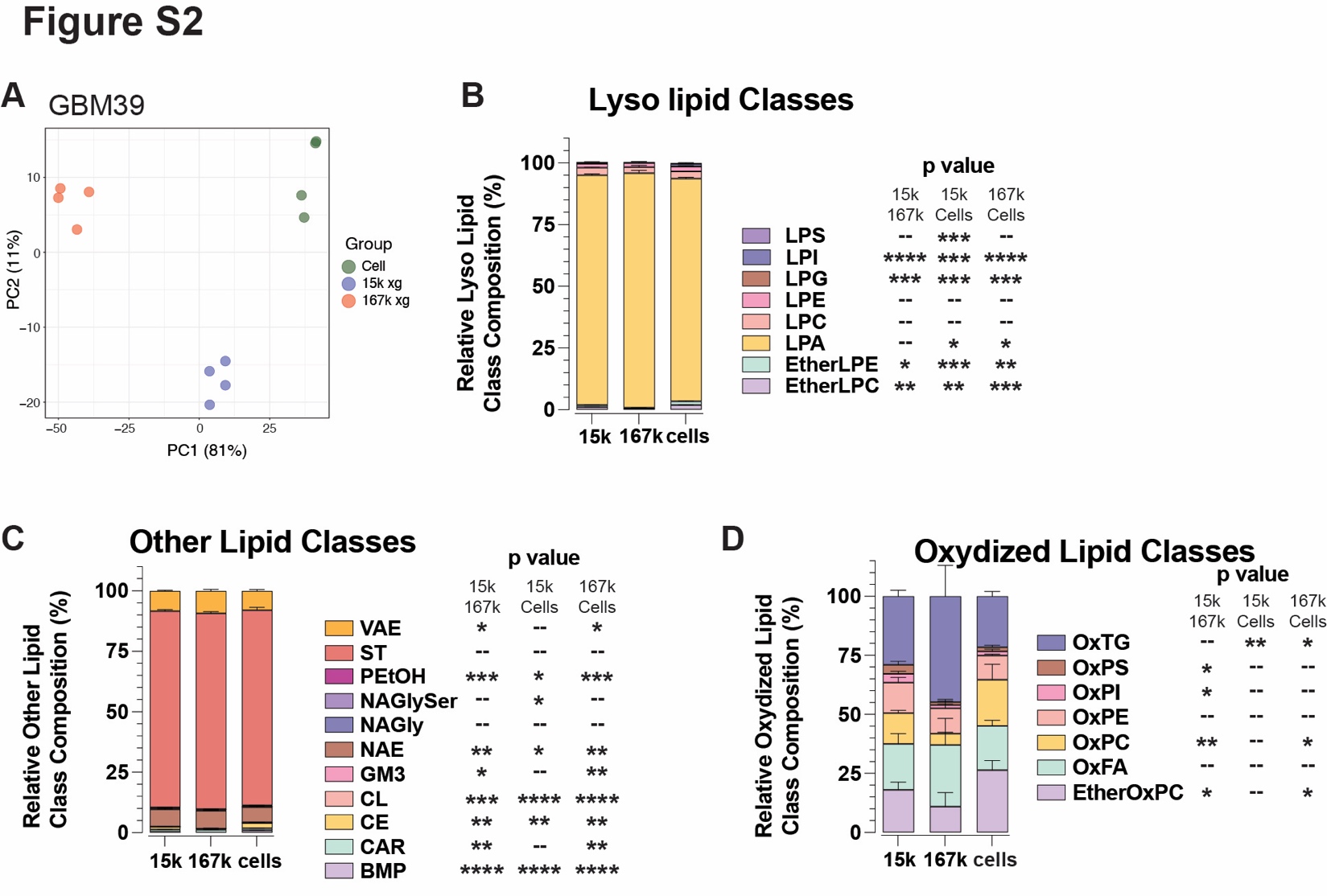


**Fig. S2. Lipid classes and subclasses differ between 15k and 167k EV populations. A)** Principal component analysis (PCA) of cells and the 15k and 167k EV population samples from GBM39**. B-D)** Relative lipid subclasses composition (percentage) for lyso lipids (B), other lipids (C), and oxidized lipids (D). Data are presented as the mean ± S.E.M. of biologically independent replicates (n=4), unpaired t test, two-tailed, *p<0.05, **p<0.01, ***p<0.001 and ****p<0.0001.


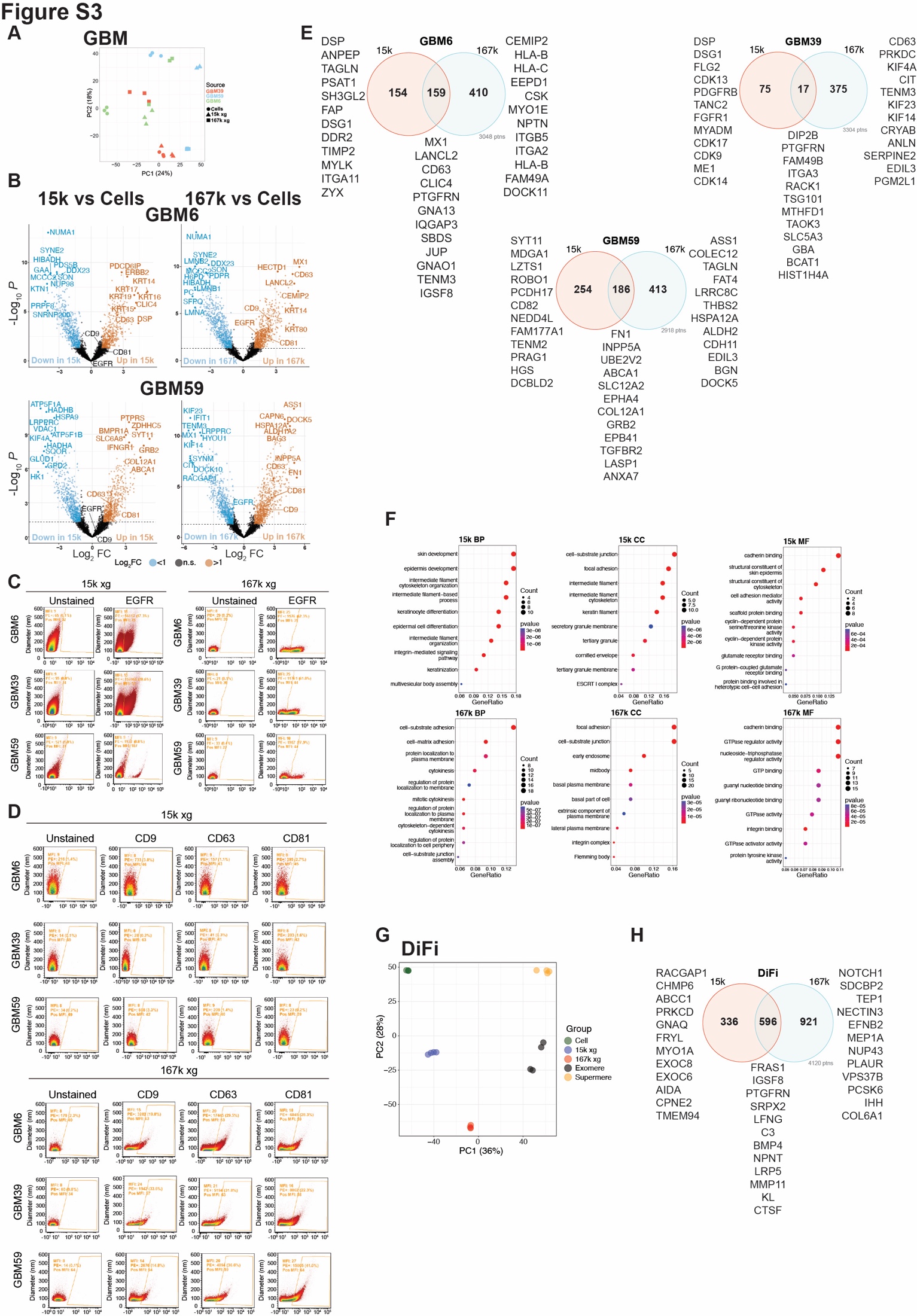


**Fig. S3. Preferential loading of proteins in 15k and 167k EVs.** **A)** PCA of cells, 15k and 167k EV population samples from GBM6, GBM39 and GBM59**. B)** Volcano plots of proteins from GBM39 and GBM59 15k and 167k EV fractions vs cells. EGFR, CD9, CD63 and CD81 markers are highlighted. **C,D)** Representative nanoflow cytometry plots of 15k and 167k EVs isolated from GBM6, GBM39, and GBM59 stained for the indicated markers. **E)** Venn diagram of protein species preferentially present (Log2FC >1) in 15k and 167k EVs vs cells from GBM6, GBM39 and GBM59 PDX cells. The top 12 proteins that are unique to 15k and 167k EVs and common are indicated. **F)** Gene ontology (GO) analysis of Biological Processes (BP), Molecular Function (MF) and Cellular Components (CC) of the proteins in common in 2 out of 3 and 3 out of 3 of GBM6, GBM39 and GBM59 for 15k and 167k EV proteins. **G)** PCA of cells, 15k and 167k EV population samples, exomeres and supermeres from DiFi cells. **H)** Venn diagram of protein species preferentially present (Log2FC >1) in 15k and 167k EVs vs cells from DiFi cells. The top 12 proteins that are unique to 15k and 167k EVs and common are indicated.


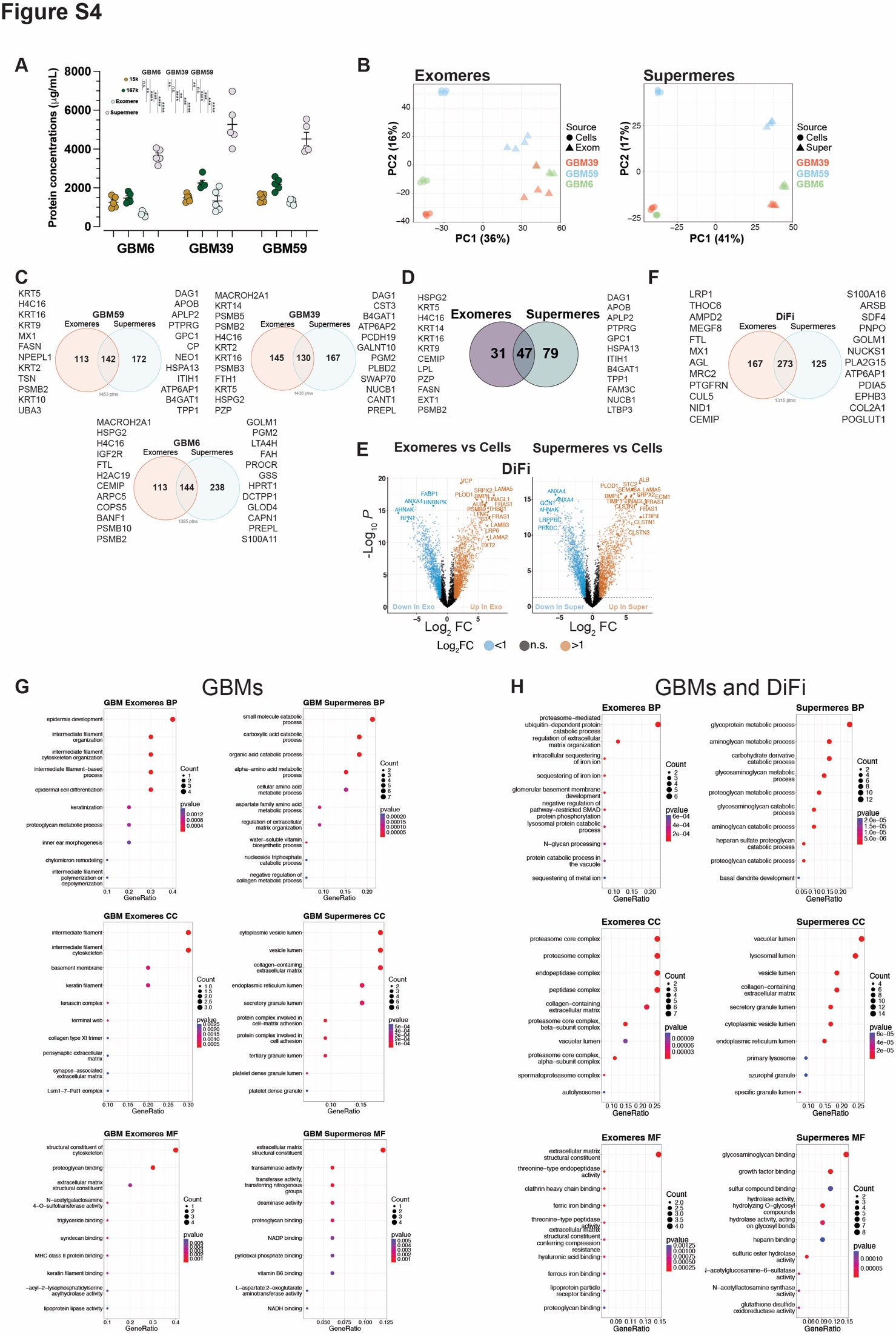


**Fig. S4. Proteomes of exomeres and supermeres. A)** Protein concentrations of 15k EVs, 167k EVs, exomeres and supermeres from GBM6, GBM39 and GBM59. **B)** PCA of cells, exomeres and supermeres for GBM6, GBM39 and GBM59. **C)** Venn diagram of protein species preferentially present (Log2FC >1) in exomeres and supermeres vs cells from GBM6, GBM39, and GBM59. The top 12 proteins that are unique to exomeres and supermeres are indicated. **D)** Venn diagram of overlap between exomeres and supermeres from all three GBM PDX lines. Indicated are the top 12 highest expressed exomere-specific and supermere-specific proteins. **E)** Volcano plots of proteins from DiFi exomeres and supermeres vs cells. **F)** Venn diagram of DiFi exomere and supermere proteins. Indicated are the top 12 highest expressed exomere-specific and supermere-specific proteins.

**G)** GO analysis of Biological Processes (BP), Molecular Function (MF) and Cellular Components (CC) of the proteins in common in GBM6, GBM39 and GBM59 but not DiFi for exomeres (10 proteins) and supermeres (33 proteins). **H)** GO analysis of Biological Processes (BP), Molecular Function (MF) and Cellular Components (CC) of the proteins in common in GBM6, GBM39, GBM59 and DiFi for exomeres (29 proteins) and supermeres (54 proteins).


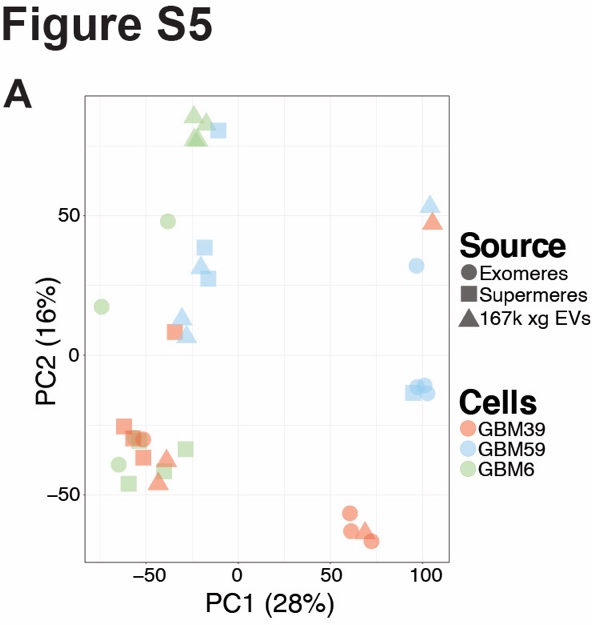


**Fig. S5. Unique lipid composition amongst exomeres, supermeres and 167k EVs. A)** Principal component analysis (PCA) of lipidomes from 167k EV populations, exomeres and supermeres isolated from GBM6, GBM39 and GBM59.


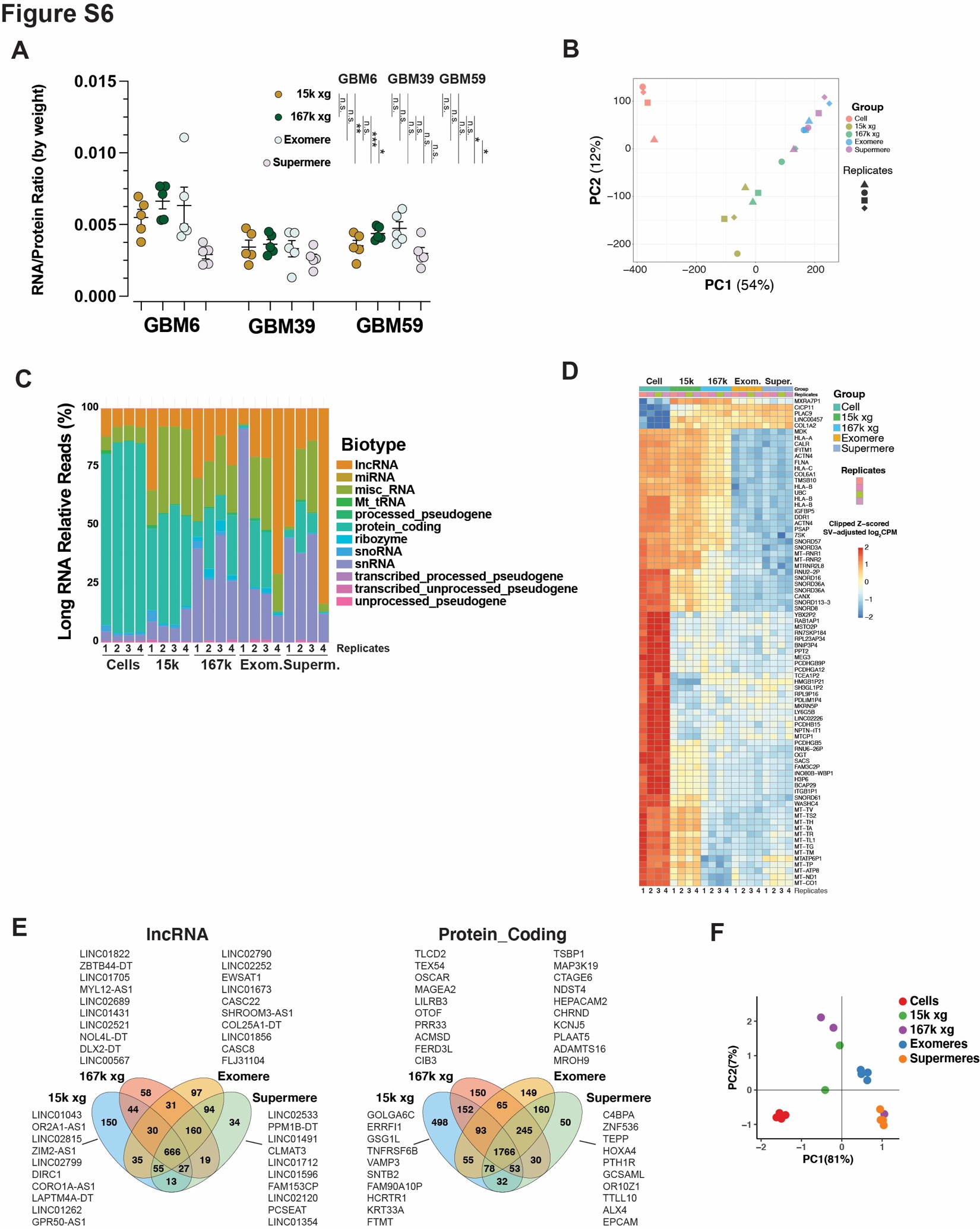


**Fig. S6.** **Distinct abundance of long- and small-exRNAs in extracellular compartments. A)** Levels of RNA isolated from 15k EVs, 167k EVs, exomeres and supermeres from GBM6, GBM39 and GBM59 PDX cells normalized to protein levels from the same compartments. Data are presented as the mean ± S.E.M. of biologically independent replicates (n=5), unpaired t test, two-tailed, *p<0.05, **p<0.01, and ***p<0.001. **B)** Principal component (PC) analysis of normalized RNA reads isolated from biologically independent replicates (n=4) of cells and the indicated extracellular compartments from GBM39 PDX cells. **C)** Percentage of long-exRNA reads mapped to indicated biotypes from GBM39 PDX cells, 15k EVs, 167k EVs, exomeres and supermeres. lncRNA, long non-coding RNA; miRNA, microRNA; misc_RNA, miscellaneous RNA; Mt_tRNA; mitochondrial tRNA; snoRNA, small nucleolar RNA; rRNA, ribosomal RNA; n=4 independent samples. **D)** Heatmap of the top-10 most abundant long-RNAs across GBM39 PDX cells and extracellular compartments. **E)** Venn diagram of unique and common lncRNA and protein_coding long-RNAs and top-10 most differentially abundant (when compared to cellular levels) in 15k EVs, 167k EVs, exomere and supermere fractions isolated from GBM39 PDX cells unique to each extracellular compartments. Numbers inside diagrams represent the number of lncRNA and protein_coding that displays a log2FC >1 when compared to cells. **F)** PCA of normalized RNA reads isolated from biologically independent replicates (n=2-4) of cells and the indicated extracellular compartments from GBM39 PDX cells.


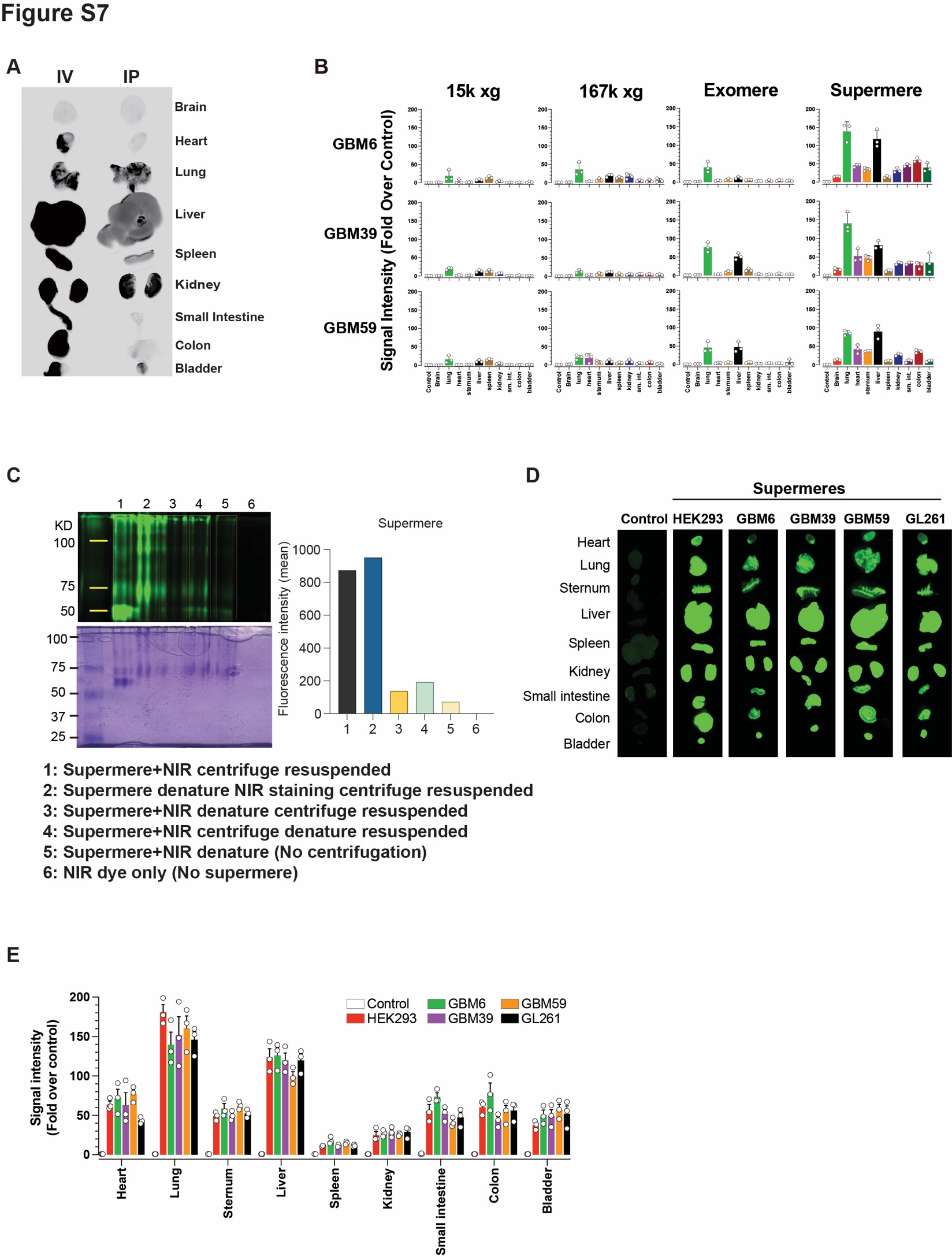


**Fig. S7. Biodistribution of EVs and NVEPs in vivo. A)** Whole-organ imaging. Male CD-1 mice were injected intraperitoneally (IP) or intravenously (IV) via the tail-vein with 150 μg (protein) (in 200 mL of PBS) UC-isolated, NIR-labelled GBM39 PDX cells-derived supermeres. The organs were harvested 6 hr post injection and analyzed. **B)** Quantitation of organ imaging. Data are presented as the mean ± S.E.M. of biologically independent replicates (n=3). **C)** NIR signal detection (top left) and signal intensity quantitation (right panel) and Coomassie-blue staining of SDS-PAGE (bottom left) of UC-isolated GBM39 supermeres treated under the indicated denaturation steps. **D,E)** Whole-organ imaging. Representative NIR images of organs (D) and quantitation (E) of NIR-labeled GBM6, GBM39 , GBM59, GL261 and HEK-293 supermeres. Mice (n=3) were injected (IV) with 150 μg (protein) (in 200 mL of PBS).


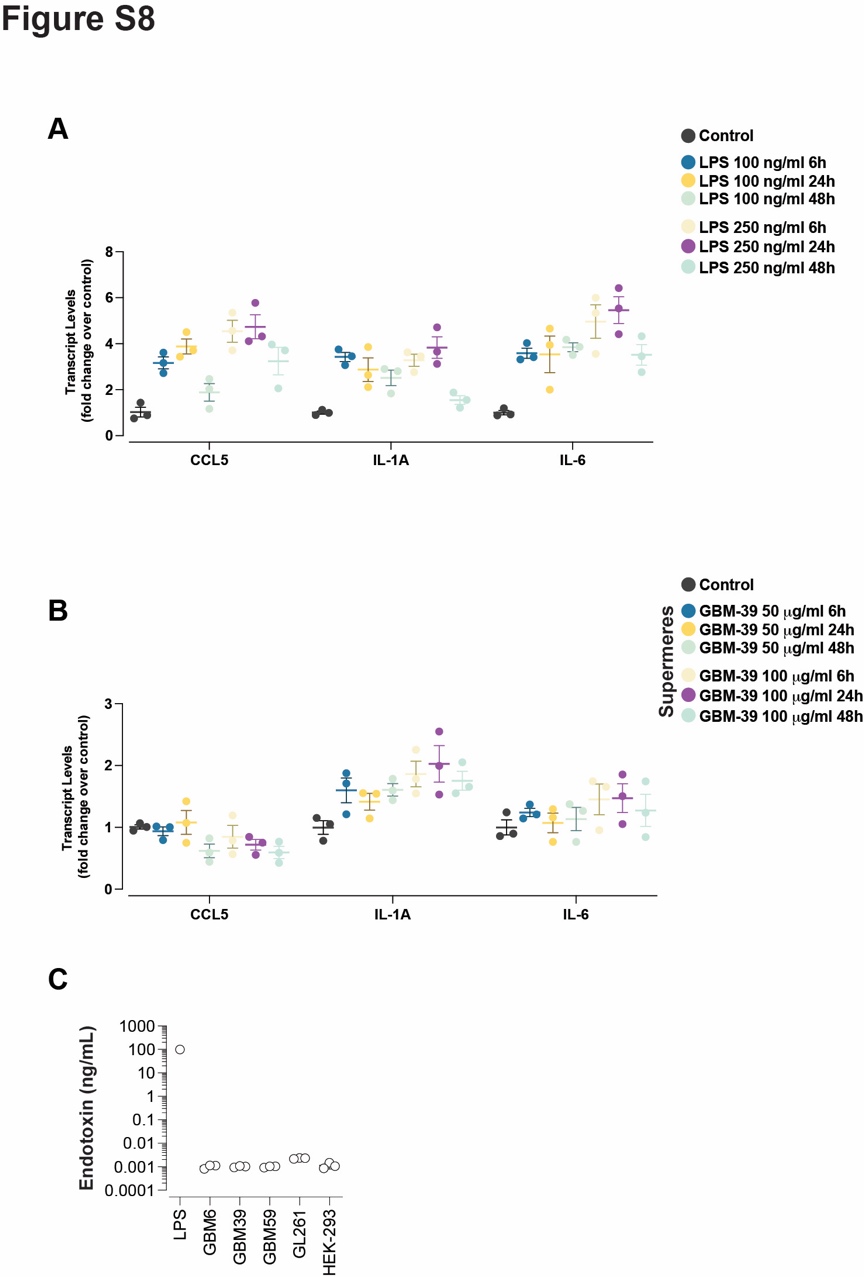


**Fig. S8. Dose and Time response of LPS and supermere stimulation.** **A,B)** qRT PCR of the indicated genes of RNA isolated from HMC3 cells treated with LPS (100 and 250 ng/mL) (A) or supermeres from GBM39 PDX cells (50 and 100 μg/mL) for 6, 24 and 48 hr (B). Data are presented as the mean ± S.E.M. of biologically independent replicates (n=3), unpaired t test, two-tailed. Unless otherwise indicated, pairwise comparisons within a series (6hr vs 24 hr vs 48 hr) or in between two series (low vs high concentrations) are non-significant. **C)** Endotoxin chromogenic LAL (Limulus Amebocyte Lysate) assay of 5 μg of supermeres isolated from the indicated cell lines.
